# Learning Universal Computations with Spikes

**DOI:** 10.1371/journal.pcbi.1004895

**Published:** 2016-06-16

**Authors:** Dominik Thalmeier, Marvin Uhlmann, Hilbert J. Kappen, Raoul-Martin Memmesheimer

**Affiliations:** 1 Donders Institute, Department of Biophysics, Radboud University, Nijmegen, Netherlands; 2 Max Planck Institute for Psycholinguistics, Department for Neurobiology of Language, Nijmegen, Netherlands; 3 Donders Institute, Department for Neuroinformatics, Radboud University, Nijmegen, Netherlands; 4 Center for Theoretical Neuroscience, Columbia University, New York, New York, United States of America; University of Tübingen and Max Planck Institute for Biologial Cybernetics, GERMANY

## Abstract

Providing the neurobiological basis of information processing in higher animals, spiking neural networks must be able to learn a variety of complicated computations, including the generation of appropriate, possibly delayed reactions to inputs and the self-sustained generation of complex activity patterns, e.g. for locomotion. Many such computations require previous building of intrinsic world models. Here we show how spiking neural networks may solve these different tasks. Firstly, we derive constraints under which classes of spiking neural networks lend themselves to substrates of powerful general purpose computing. The networks contain dendritic or synaptic nonlinearities and have a constrained connectivity. We then combine such networks with learning rules for outputs or recurrent connections. We show that this allows to learn even difficult benchmark tasks such as the self-sustained generation of desired low-dimensional chaotic dynamics or memory-dependent computations. Furthermore, we show how spiking networks can build models of external world systems and use the acquired knowledge to control them.

## Author Summary

Animals and humans can learn versatile computations such as the generation of complicated activity patterns to steer movements or the generation of appropriate outputs in response to inputs. Such learning must be accomplished by networks of nerve cells in the brain, which communicate with short electrical impulses, so-called spikes. Here we show how such networks may perform the learning. We track their ability back to experimentally found nonlinearities in the couplings between nerve cells and to a network connectivity that complies with constraints. We show that the spiking networks are able to learn difficult tasks such as the generation of desired chaotic activity and the prediction of the impact of actions on the environment. The latter allows to compute optimal actions by mental exploration.

## Introduction

The understanding of neural network dynamics on the mesoscopic level of hundreds and thousands of neurons and their ability to learn highly complicated computations is a fundamental open challenge in neuroscience. For biological systems, such an understanding will allow to connect the microscopic level of single neurons and the macroscopic level of cognition and behavior. In artificial computing, it may allow to propose new, possibly more efficient computing schemes.

Randomly connected mesoscopic networks can be a suitable substrate for computations [1–5], as they reflect the input in a complicated, nonlinear way and at the same time maintain, like a computational “reservoir”, fading memory of past inputs as well as of transformations and combinations of them. This includes the results of computations on current and past inputs. Simple readout neurons may then learn to extract the desired result; the computations are executed in real time, i.e. without the need to wait for convergence to an attractor (“reservoir computing”) [1, 2]. Non-random and adaptive network connectivity can change performance [6–8].

Networks with higher computational power, in particular with the additional ability to learn self-sustained patterns of activity and persistent memory, require an output feedback or equivalent learning of their recurrent connections [2, 3]. However, network modeling approaches achieving such universal (i.e. general purpose) computational capabilities so far concentrated on networks of continuous rate units [2, 4], which do not take into account the characteristics that neurons in biological neural networks communicate via spikes. Indeed, the dynamics of spiking neural networks are discontinuous, usually highly chaotic, variable, and noisy. Readouts of such spiking networks show low signal-to-noise ratios. This hinders computations following the described principle in particular in presence of feedback or equivalent plastic recurrent connections, and has questioned it as model for computations in biological neural systems [9–11].

Here we first introduce a class of recurrent spiking neural networks that are suited as a substrate to learn universal computations. They are based on standard, established neuron models, take into account synaptic or dendritic nonlinearities and are required to respect some structural constraints regarding the connectivity of the network. To derive them we employ a precise spike coding scheme similar to ref. [12], which was introduced to approximate linear continuous dynamics.

Thereafter we endow the introduced spiking networks with learning rules for either the output or the recurrent connection weights and show that this enables them to learn equally complicated, memory dependent computations as non-spiking continuous rate networks. The spiking networks we are using have only medium sizes, between tens and a few thousands of neurons, like networks of rate neurons employed for similar tasks. We demonstrate the capabilities of our networks by applying them to challenging learning problems which are of importance in biological contexts. In particular, we show how spiking neural networks can learn the self-sustained generation of complicated dynamical patterns, and how they can build world models, which allow to compute optimal actions to appropriately influence an environment.

## Results

### Continuous signal coding spiking neural networks (CSNs)

#### Network architecture

For our study, we use leaky integrate-and-fire neurons. These incorporate crucial features of biological neurons, such as operation in continuous time, spike generation and reset, while also maintaining some degree of analytical tractability. A network consists of *N* neurons. The state of a neuron *n* is given by its membrane potential *V*_*n*_(*t*). The membrane potential performs a leaky integration of the input and a spike is generated when *V*_*n*_(*t*) reaches a threshold, resulting in a spiketrain

$$s_{n}\left(t\right)=\sum_{t_{n}}\delta \left(t-t_{n}\right)$$ (1)

with spike times *t*_*n*_ and the Dirac delta-distribution *δ*. After a spike, the neuron is reset to the reset potential, which lies *θ* below the threshold. The spike train generates a train of exponentially decaying normalized synaptic currents

$$r_{n}\left(t\right)=\sum_{t_{n}}e^{-\lambda_{s}\left(t-t_{n}\right)}\Theta \left(t-t_{n}\right)\;\;\Leftrightarrow \;\;{\dot{r}}_{n}\left(t\right)=-\lambda_{s}r_{n}\left(t\right)+s_{n}\left(t\right),$$ (2)

where $\tau_{s}=\lambda_{s}^{-1}$ is the time constant of the synaptic decay and Θ(.) is the Heaviside theta-function.

**Fig 1:**
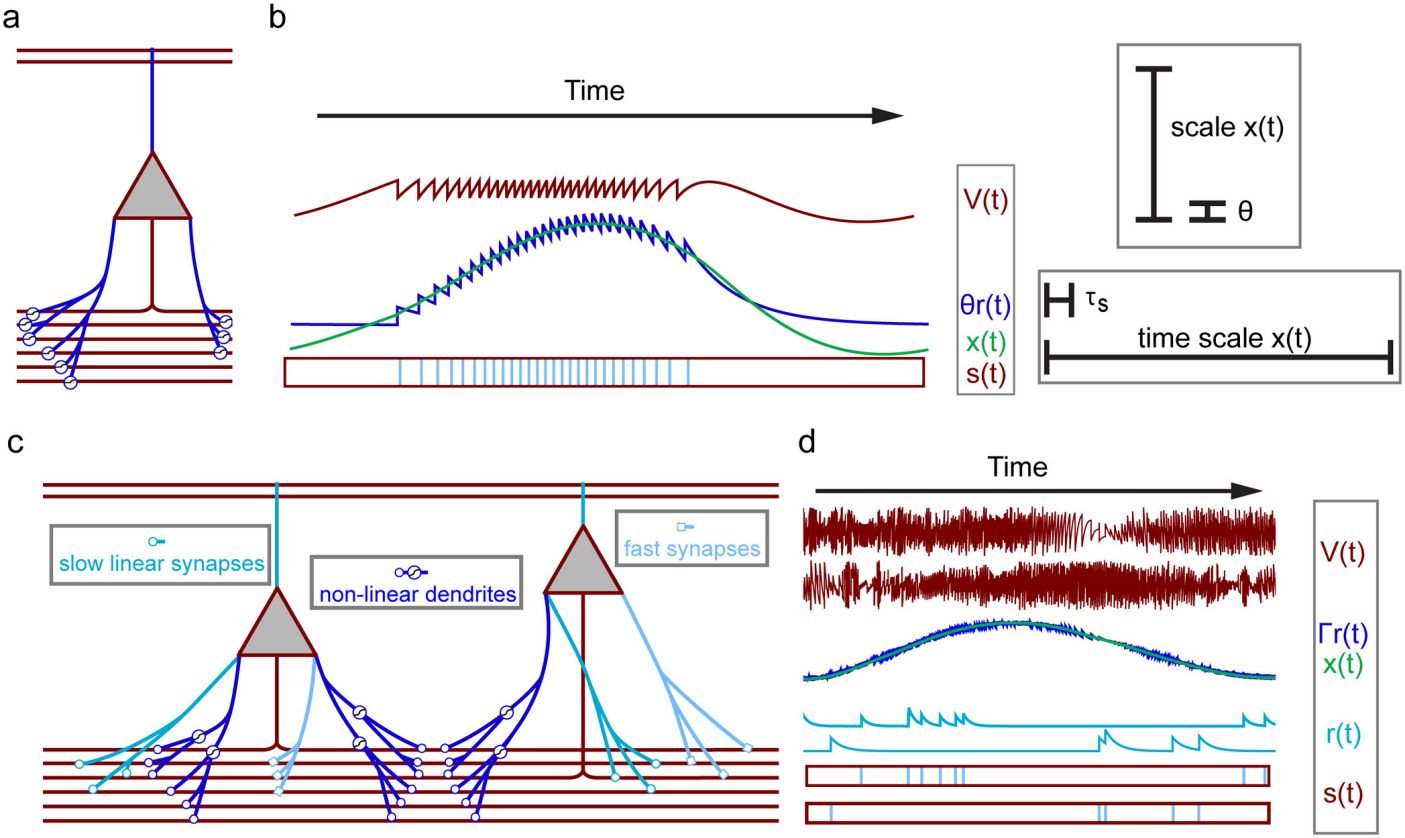
Coding of continuous signals in neurons with saturating synapses (a,b) and nonlinear dendrites (c,d). (a,b): A neuron with saturating synapses (a) that directly codes for a continuous signal (b). Panel (a) displays the neuron with an axon (red) and dendrites (dark blue) that receive inputs from the axons of other neurons (axons at the bottom) via saturating synapses (symbolized by sigmoids at the synaptic contacts). The currents entering the soma are weighted sums of input spike trains that are synaptically filtered (generating scaled normalized synaptic currents *γr*_*n*_(*t*), synaptic time scale *τ*_*s*_) and thereafter subject to a saturating synaptic nonlinearity. External inputs (axons at the top) are received without saturation. The continuous signal *x*(*t*) (panel b left hand side, green) is the sum of the neuron’s membrane potential *V*(*t*) (red) and its scaled normalized synaptic current *θr*(*t*) (dark blue). *r*(*t*) is a low-pass filtered version of the neuron’s spike train *s*(*t*) (light blue in red box). If *x*(*t*)>0, the time scale of *x*(*t*) should be large against the synaptic time scale *τ*_*s*_ and *x*(*t*) should predominantly be large against the neuron’s threshold, *θ*2 (panel b right hand side, assumptions 1,2 in the main text). *x*(*t*) is then already well approximated by *θr*(*t*), while *V*(*t*) is oscillating between ±*θ*2. If *x*(*t*)≤0, we have *V*(*t*)≤0, no spikes are generated and *r*(*t*) quickly decays to zero, such that we predominantly have *r*(*t*)≈0 and *x*(*t*) is well approximated by *V*(*t*) (cf. Eq (9)). (c,d): Two neurons with nonlinear dendrites (c) from a larger network that distributedly codes for a continuous signal (d). (c): Each neuron has an axon (red) and different types of dendrites (cyan, light blue and dark blue) that receive inputs from the axons of other neurons (axons at the bottom) via fast or slow conventional synapses (highlighted by circles and squares). Linear dendrites with slow synapses (cyan with circle contacts) generate somatic currents that are weighted linear sums of low-pass filtered presynaptic spike trains (weighted sums of the *r*_*n*_(*t*)). Linear dendrites with fast synapses (light blue with square contacts) generate somatic currents with negligible filtering (weighted sums of the spike trains *s*_*n*_(*t*)). Spikes arriving at a nonlinear dendrite (dark blue) are also filtered (circular contact). The resulting *r*_*n*_(*t*) are weighted, summed up linearly in the dendrite and subjected to a saturating dendritic nonlinearity (symbolized by sigmoids at dendrites), before entering the soma. We assume that the neurons have nonlinear dendrites that are located in similar tissue areas, such that they connect to the same sets of axons and receive similar inputs. (d): All neurons in the network together encode *J* continuous signals **x**(*t*) (one displayed in green) by a weighted sum of their membrane potentials **V**(*t*) (two traces of different neurons displayed in red) and their normalized PSCs **r**(*t*) (two traces displayed in cyan). The **Γr**(*t*) alone already approximate **x**(*t*) well. The neurons’ output spike trains **s**(*t*) (light blue in red box) generate slow and fast inputs to other neurons. (Note that spikes can be generated due to suprathreshold excitation by fast inputs. Since we plot **V**(*t*) after fast inputs and possible resets, the corresponding threshold crossings do not appear.)

Throughout the article we consider two closely related types of neurons, neurons with saturating synapses and neurons with nonlinear dendrites (cf. Fig 1). In the model with saturating synapses (Fig 1a), the membrane potential *V*_*n*_(*t*) of neuron *n* obeys

$$\begin{array}{ll} {\dot{V}}_{n}\left(t\right)= & -\lambda_{V}V_{n}\left(t\right)+\overset{N}{\underset{m=1}{\sum }}A_{nm}\tanh\left(\gamma r_{m}\left(t\right)\right)+V_{\text{r}}\lambda_{s}r_{n}\left(t\right)\\ & -\theta s_{n}\left(t\right)+I_{\text{e,}n}\left(t\right), \end{array}$$ (3)

with membrane time constant $\tau_{m}=\lambda_{V}^{-1}$.

The saturation of synapses, e.g. due to receptor saturation or finite reversal potentials, acts as a nonlinear transfer function [13, 14], which we model as a tanh-nonlinearity (since *r*_*m*_(*t*)≥0 only the positive part of the tanh becomes effective). We note that this may also be interpreted as a simple implementation of synaptic depression: A spike generated by neuron *m* at *t*_*m*_ leads to an increase of *r*_*m*_(*t*_*m*_) by 1. As long as the synapse connecting neuron *m* to neuron *n* is far from saturation (linear part of the tanh-function) this leads to the consumption of a fraction *γ* of the synaptic “resources” and the effect of the spike on the neuron is approximately the effect of a current *A*_*nm*_ *γe*^−λ_*s*_(*t* − *t*_*m*_)^ Θ(*t* − *t*_*m*_). When a larger number of such spikes arrive in short time such that the consumed resources accumulate to 1 and beyond, the synapse saturates at its maximum strength *A*_*nm*_ and the effect of individual inputs is much smaller than before. The recovery from depression is here comparably fast, it takes place on a timescale of $\lambda_{s}^{-1}$ (compare, e.g., [15]).

The reset of the neuron is incorporated by the term −*θs*_*n*_(*t*). The voltage lost due to this reset is partially recovered by a slow recovery current (afterdepolarization) *V*_r_ λ_*s*_ *r*_*n*_(*t*); its temporally integrated size is given by the parameter *V*_r_. This is a feature of many neurons e.g. in the neocortex, in the hippocampus and in the cerebellum [16], and may be caused by different types of somatic or dendritic currents, such as persistent and resurgent sodium and calcium currents, or by excitatory autapses [17, 18]. It provides a simple mechanism to sustain (fast) spiking and generate bursts, e.g. in response to pulses. *I*_e,*n*_(*t*) is an external input, its constant part may be interpreted as sampling slow inputs specifying the resting potential that the neuron asymptotically assumes for long times without any recurrent network input. We assume that the resting potential is halfway between the reset potential *V*_res_ and the threshold *V*_res_ + *θ*. We set it to zero such that the neuron spikes when the membrane potential reaches *θ*2 and resets to −*θ*2. To test the robustness of the dynamics we sometimes add a white noise input *η*_*n*_(*t*) satisfying $\langle \eta_{n}\left(t\right)\eta_{m}\left(t^{\prime }\right)\rangle =\sigma_{\eta }^{2}\delta_{nm}\delta \left(t-t^{\prime }\right)$ with the Kronecker delta *δ*_*nm*_.

For simplicity, we take the parameters λ_*V*_,*θ*,*V*_r_ and *γ*,λ_*s*_ identical for all neurons and synapses, respectively. We take the membrane potential *V*_*n*_ and the parameters *V*_r_ and *θ* dimensionless, they can be fit to the voltage scale of biological neurons by rescaling with an additive and a multiplicative dimensionful constant. Time is measured in seconds.

We find that networks of the form Eq (3) generate dynamics suitable for universal computation similar to continuous rate networks [2, 4], if 0 < λ_*x*_ ≪ λ_*s*_, where $\lambda_{x}=\lambda_{s}(1-\frac{V_{\text{r}}}{\theta })$, *A*_*nm*_ sufficiently large and *γ* small. The conditions result from requiring the network to approximate a nonlinear continuous dynamical system (see next section).

An alternative interpretation of the introduced nonlinearity is that the neurons have nonlinear dendrites, where each nonlinear compartment is small such that it receives at most one (conventional, nonsaturating) synapse. *A*_*nm*_ is then the strength of the coupling from a dendritic compartment to the soma. This interpretation suggests an extension of the neuron model allowing for several dendrites per neuron, where the inputs are linearly summed up and then subjected to a saturating dendritic nonlinearity [19–21]. Like the previous model, we find that such a model has to satisfy additional constraints to be suitable for universal computation:

Neurons with nonlinear dendrites need additional slow and fast synaptic contacts which arrive near the soma and are summed linearly there (Fig 1c). Such structuring has been found in biological neural networks [22]. We gather the different components into a dynamical equation for *V*_*n*_ as

$$\begin{array}{ll} {\dot{V}}_{n}\left(t\right)= & -\lambda_{V}V_{n}\left(t\right)+\overset{J}{\underset{j=1}{\sum }}D_{nj}\tanh\left(\overset{N}{\underset{m=1}{\sum }}W_{njm}r_{m}\left(t\right)\right)\\ & +\overset{N}{\underset{m=1}{\sum }}{\tilde{U}}_{nm}r_{m}\left(t\right)-\overset{N}{\underset{m=1}{\sum }}U_{nm}s_{m}\left(t\right)\\ & +\overset{J}{\underset{j=1}{\sum }}\Gamma_{jn}I_{\text{e},j}\left(t\right). \end{array}$$ (4)

*D*_*nj*_ is the coupling from the *j*th dendrite of neuron *n* to its soma. The total number of dendrites and neurons is referred to as *J* and *N* respectively. *W*_*njm*_ is the coupling strength from neuron *m* to the *j*th nonlinear dendrite of neuron *n*. The slow, significantly temporally filtered inputs from neuron *m* to the soma of neuron *n*, ${\tilde{U}}_{nm}r_{m}\left(t\right)$, have connection strengths ${\tilde{U}}_{nm}$. The fast ones, *U*_*nm*_ *s*_*m*_(*t*), have negligible synaptic filtering (i.e. negligible synaptic rise and decay times) as well as negligible conduction delays. The resets and recoveries are incorporated as diagonal elements of the matrices *U*_*nm*_ and ${\tilde{U}}_{nm}$. To test the robustness of the dynamics, also here we sometimes add a white noise input *η*_*n*_(*t*). To increase the richness of the recurrent dynamics and the computational power of the network (cf. [23] for disconnected units without output feedback) we added inhomogeneity, e.g. through the external input current in some tasks. In the control/mental exploration task, we added a constant bias term *b*_*j*_ as argument of the tanh to introduce inhomogeneity.

We find that the network couplings **D**,**W**,**U** and $\tilde{U}$ Eq (4) (we use bold letters for vectors and matrices) should satisfy certain interrelations. As motivated in the subsequent section and derived in the supporting material, their components may be expressed in terms of the components of a *J*×*N* matrix **Γ**, and a *J*×*J* matrix **A** as $D_{nj}=\sum_{i=1}^{J}\Gamma_{in}A_{ij}$, *W*_*njm*_ = *Γ*_*jm*_, ${\tilde{U}}_{nm}=a\sum_{j=1}^{J}\Gamma_{jn}\Gamma_{jm}+\mu \lambda_{s}\delta_{nm}$, $U_{nm}=\sum_{j=1}^{J}\Gamma_{jn}\Gamma_{jm}+\mu \delta_{nm}$, where *a* = λ_*s*_ − λ_*x*_ and *μ* ≥ 0 is small (see also Table 1 for an overview). The thresholds are chosen identical, *θ*^*n*^ = *θ*, see Methods.

**Table 1:** Parameters of a network of neurons with nonlinear dendrites (cf. Eq (4)) and their optimal values.

	explanation	optimal value
*D*_*nj*_	coupling from the *j*th dendrite of neuron *n*to its soma	$D_{nj}=\sum_{i=1}^{J}\Gamma_{in}A_{ij}$
*W*_*njm*_	coupling strength from neuron *m* to the *j*th nonlinear dendrite of neuron *n*	*W*_*njm*_ = *Γ*_*jm*_
${\tilde{U}}_{nm}$	slow coupling from neuron *m* to neuron *n*; diagonal elements incorporate a recovery current	${\tilde{U}}_{nm}=a\sum_{j=1}^{J}\Gamma_{jn}\Gamma_{jm}+\mu \lambda_{s}\delta_{nm}$ *a* = λ_*s*_ − λ_*x*_
*U*_*nm*_	fast coupling from neuron *m* to neuron *n*; diagonal elements incorporate the reset	$U_{nm}=\sum_{j=1}^{J}\Gamma_{jn}\Gamma_{jm}+\mu \delta_{nm}$
*θ*^*n*^	threshold of neuron *n*	$\theta^{n}=\frac{U_{nn}}{2}$

Again, the conditions result from requiring the network to approximate a nonlinear continuous dynamical system. This system, Eq (11), is characterized by the *J*×*J* coupling matrix **A** and a *J*-dimensional input **c**(*t*) whose components are identical to the *J* independent components of the external input current **I**_e_ in Eq (4); the matrix **Γ** is a decoding matrix that fixes the relation between spiking and continuous dynamics (see next section). We note that the matrices **Γ** and **A** are largely unconstrained, such that the coupling strengths maintain a large degree of arbitrariness. Ideally, *W*_*njm*_ is independent of *n*, therefore neurons have dendrites that are similar in their input characteristics to dendrites in some other neurons (note that **D** may have zero entries, so dendrites can be absent). We interpret these as dendrites that are located in a similar tissue area and therefore connect to the same axons and receive similar inputs (cf. Fig 1c for an illustration). The interrelations between the coupling matrices might be realized by spike-timing dependent synaptic or structural plasticity. Indeed, for a simpler model and task, appropriate biologically plausible learning rules have been recently highlighted [24, 25]. We tested robustness of our schemes against structural perturbations (see Figs C and D in S1 Text), in particular for deviations from the *n*-independence of *W*_*njm*_ (Fig C in S1 Text).

The networks Eq (3) with saturating synapses have a largely unconstrained topology, in particular they can satisfy the rule that neurons usually act only excitatorily or inhibitorily. For the networks Eq (4) with nonlinear dendrites, it is less obvious how to reconcile the rule with the constraints on the network connectivity. Solutions for this have been suggested in simpler systems and are subject to current research [12].

The key property of the introduced neural architecture is that the spike trains generated by the neurons encode with high signal-to-noise ratio a continuous signal that can be understood in terms of ordinary differential equations. In the following section we show how this signal is decoded from the spike trains. Thereafter, we may conclude that the spiking dynamics are sufficiently “tamed” such that standard learning rules can be applied to learn complicated computations.

#### Direct encoding of continuous dynamics

The dynamics of a neural network with *N* integrate-and-fire neurons consist of two components, the sub-threshold dynamics **V**(*t*) = (*V*_1_(*t*),…,*V*_*N*_(*t*))^*T*^ of the membrane potentials and the spike trains **s**(*t*) = (*s*_1_(*t*),…,*s*_*N*_(*t*))^*T*^ (Eq (1)), which are temporal sequences of *δ*-distributions. In the model with saturating synapses, all synaptic interactions are assumed to be significantly temporally filtered, such that the *V*_*n*_(*t*) are continuous except at reset times after spiking (Eq (3)). We posit that the **V**(*t*) and the **s**(*t*) should together form some *N*-dimensional continuous dynamics **x**(*t*) = (*x*_1_(*t*),…,*x*_*N*_(*t*))^*T*^. The simplest approach is to setup **x**(*t*) as a linear combination of the two components **V**(*t*) and **s**(*t*). To avoid infinities in *x*_*n*_(*t*), we need to eliminate the occurring *δ*-distributions by employing a smoothed version of *s*_*n*_(*t*). This should have a finite discontinuity at spike times such that the discontinuity in *V*_*n*_(*t*) can be balanced. A straightforward choice is to use *θr*_*n*_(*t*) (Eq (2)) and to set

$$V_{n}\left(t\right)+\theta r_{n}\left(t\right)=x_{n}\left(t\right)$$ (5)

(cf. Fig 1b). When the abovementioned conditions on λ_*x*_, λ_*s*_, **A** and *γ* are satisfied (cf. end of the section introducing networks with saturating synapses), the continuous signal **x**(*t*) follows a system of first order nonlinear ordinary differential equations similar to those describing standard non-spiking continuous rate networks used for computations (cf. [2, 4, 26] and Eq (11) below),

$${\dot{x}}_{n}\left(t\right)=-\lambda_{V}{\left[x_{n}\left(t\right)\right]}_{-}-\lambda_{x}{\left[x_{n}\left(t\right)\right]}_{+}+\sum_{m=1}^{N}A_{nm}\tanh\left(\frac{\gamma }{\theta }{\left[x_{m}\left(t\right)\right]}_{+}\right)+I_{\text{e},n}\left(t\right),$$ (6)

with the rectifications [*x*_*n*_(*t*)]_+_ = max(*x*_*n*_(*t*),0), [*x*_*n*_(*t*)]_−_ = min(*x*_*n*_(*t*),0). We call spiking networks where this is the case *continuous signal coding spiking neural networks (CSNs)*.

Except for the rectifications, Eq (6) has a standard form for non-spiking continuous rate networks, used for computations [2, 4, 26]. A salient choice for λ_*x*_ is λ_*x*_ = λ_*V*_, i.e. $V_{\text{r}}=(1-\frac{\lambda_{V}}{\lambda_{s}})\theta$, such that the rectifications outside the tanh-nonlinearity vanish. Eq (6) generates dynamics that are different from the standard ones in the respect that the trajectories of individual neurons are, e.g. for random Gaussian matrices **A**, not centered at zero. However, they can satisfy the conditions for universal computation (enslaveability/echo state property and high dimensional nonlinear dynamics) and generate longer-term fading memory for appropriate scaling of **A**. Also the corresponding spiking networks are then suitable for fading memory-dependent computations. Like for the standard networks [27, 28], we can derive sufficient conditions to guarantee that the dynamics Eq (6) are enslaveable by external signals (echo state property). ∥**A**∥ < min(*λ_V_*, *λ_x_*), where ∥**A**∥ is the largest singular value of the matrix **A**, provides such a condition (see Supplementary material for the proof). The condition is rather strict, our applications indicate that the CSNs are also suited as computational reservoirs when it is violated. This is similar to the situation in standard rate network models [27]. We note that if the system is enslaved by an external signal, the time scale of *x*_*n*_(*t*) is largely determined by this signal and not anymore by the intrinsic scales of the dynamical system.

We will now show that spiking neural networks Eq (3) can encode continuous dynamics Eq (6). For this we derive the dynamical equation of the membrane potential Eq (3) from the dynamics of **x**(*t*) using the coding rule Eq (5), the dynamical Eq (2) for *r*_*n*_(*t*) and the rule that a spike is generated whenever *V*_*n*_(*t*) reaches threshold *θ*2: We first differentiate Eq (5) to eliminate ${\dot{x}}_{n}\left(t\right)$ from Eq (6) and employ Eq (2) to eliminate ${\dot{r}}_{n}\left(t\right)$. The resulting expression for ${\dot{V}}_{n}\left(t\right)$ reads

$${\dot{V}}_{n}\left(t\right)=-\lambda_{V}{\left[x_{n}\left(t\right)\right]}_{-}-\lambda_{x}{\left[x_{n}\left(t\right)\right]}_{+}+\sum_{m=1}^{N}A_{nm}\tanh\left(\frac{\gamma }{\theta }{\left[x_{m}\left(t\right)\right]}_{+}\right)-\theta s_{n}\left(t\right)+\lambda_{s}\theta r_{n}\left(t\right)+I_{\text{e,}n}\left(t\right).$$ (7)

It already incorporates the resets of size *θ* (cf. the term −*θs*_*n*_(*t*)), they arise since *x*_*n*_(*t*) = *V*_*n*_(*t*) + *θr*_*n*_(*t*) is continuous and *r*_*n*_(*t*) increases by one at spike times (thus V must decrease by *θ*). We now eliminate the occurrences of [*x*_*n*_(*t*)]_+_ and [*x*_*n*_(*t*)]_−_.

For this, we make two assumptions (cf. Fig 1b) on the *x*_*n*_(*t*) if they are positive:

1. The dynamics of *x*_*n*_(*t*) are slow against the synaptic timescale *τ*_*s*_,
2. the *x*_*n*_(*t*) assume predominantly values *x*_*n*_(*t*)≫*θ*2.

First we consider the case *x*_*n*_(*t*)>0. Since *V*_*n*_(*t*) is reset when it reaches its threshold value *θ*2, *V*_*n*_(*t*) is always smaller than *θ*2. Thus, given *V*_*n*_(*t*)>0 assumption 2 implies that we can approximate *x*_*n*_(*t*)≈*θr*_*n*_(*t*), as the contribution of *V*_*n*_(*t*) is negligible because *V*_*n*_(*t*)≤*θ*2. This still holds if *V*_*n*_(*t*) is negative and its absolute value is not large against *θ*2. Furthermore, assumption 1 implies that smaller negative *V*_*n*_(*t*) cannot co-occur with positive *x*_*n*_(*t*): *r*_*n*_(*t*) is positive and in the absence of spikes it decays to zero on the synaptic time scale *τ*_*s*_ (Eq (2)). When *V*_*n*_(*t*)<0, neuron *n* is not spiking anymore. Thus when *V*_*n*_(*t*) is shrinking towards small negative values and *r*_*n*_(*t*) is decaying on a timescale of *τ*_*s*_, *x*_*n*_(*t*) is also decaying on a time-scale *τ*_*s*_. This contradicts assumption 1. Thus when *x*_*n*_(*t*)>0, the absolute magnitude of *V*_*n*_(*t*) is on the order of *θ*2. With assumption 2 we can thus set *x*_*n*_(*t*)≈*θr*_*n*_(*t*), whenever *x*_*n*_(*t*)>0, neglecting contributions of size *θ*2.

Now we consider *x*_*n*_(*t*)≤0. This implies *V*_*n*_(*t*)≤0 (since always *r*_*n*_(*t*)≥0) as well as a quick decay of *r*_*n*_(*t*) to zero. When *x*_*n*_(*t*) assumes values significantly below zero, assumption 1 implies that we have *x*_*n*_(*t*)≈*V*_*n*_(*t*) and *r*_*n*_(*t*)≈0, otherwise *x*_*n*_(*t*) must have changed from larger positive (assumption 2) to larger negative values on a timescale of *τ*_*s*_.

The approximate expressions may be gathered in the replacements [*x*_*n*_(*t*)]_+_ = *θr*_*n*_(*t*) and [*x*_*n*_(*t*)]_−_ = [*V*_*n*_(*t*)]_−_. Using these in Eq (7) yields together with $\lambda_{x}=\lambda_{s}(1-\frac{V_{\text{r}}}{\theta })$

$${\dot{V}}_{n}\left(t\right)=-\lambda_{V}{\left[V_{n}\left(t\right)\right]}_{-}+\sum_{m=1}^{N}A_{nm}\tanh\left(\gamma r_{m}\left(t\right)\right)+V_{\text{r}}\lambda_{s}r_{n}\left(t\right)-\theta s_{n}\left(t\right)+I_{\text{e,}n}\left(t\right).$$ (8)

Note that our replacements allowed to eliminate the biologically implausible V-dependencies in the interaction term.

To simplify the remaining *V*_*n*_(*t*)-dependence, we additionally assume that 2’ *x*_*n*_(*t*) assumes predominantly values *x*_*n*_(*t*)≫λ_*V*_ *θ*/(2λ_*x*_), if *x*_*n*_(*t*) is positive. This can be stricter than assumption 2 depending on the values of λ_*x*_ and λ_*V*_. For positive *x*_*n*_(*t*), where λ_*V*_[*x*_*n*_(*t*)]_−_ in Eq (7) is zero, λ_*V*_ *V*_*n*_(*t*) has an absolute magnitude on the order of λ_*V*_ *θ*/2 (see the arguments above). Assumption 2’ implies that this is negligible against −λ_*x*_[*x*_*n*_(*t*)]_+_. For negative *x*_*n*_(*t*), we still have *x*_*n*_(*t*)≈*V*_*n*_(*t*). This means that we may replace −λ_*V*_[*x*_*n*_(*t*)]_−_ by λ_*V*_ *V*_*n*_(*t*) in Eq (7). Taken together, under the assumptions 1,2,2’ we may use the replacements

$$\begin{array}{c} {\left[x_{n}\left(t\right)\right]}_{+}\approx \theta r_{n}\left(t\right)\\ {\left[x_{n}\left(t\right)\right]}_{-}\approx V_{n}\left(t\right) \end{array}$$ (9)

in Eq (7), which directly yield Eq (3). Note that this also implies *r*_*n*_(*t*)≫*θ*2 if the neuron is spiking, so during active periods inter-spike-intervals need to be considerably smaller than the synaptic time scale.

Eq (6) implies that the assumptions are justified for suitable parameters: For fixed parameters $\tau_{s}=\lambda_{s}^{-1}$ and *θ* of the **r**-dynamics, we can choose sufficiently small λ_*x*_, large *A*_*nm*_ and small *γ* to ensure assumptions 1,2,2’ (cf. the conditions highlighted in the section “Network architecture”). On the other hand, for given dynamics Eq (6), we can always find a spiking system which generates the dynamics via Eqs (3), (2) and (5), and satisfies the assumptions: We only need to choose *τ*_*s*_ sufficiently small such that assumption 1 is satisfied and the spike threshold sufficiently small such that assumptions 2,2’ are satisfied. For the latter, *γ* needs to be scaled like *θ* to maintain the dynamics of *x*_*n*_ and *V*_*r*_ needs to be computed from the expression for λ_*x*_. Interestingly, we find that also outside the range where the assumptions are satisfied, our approaches can still generate good results.

The recovery current in our model has the same time constant as the slow synaptic current. Indeed, experiments indicate that they possess the same characteristic timescales: Timescales for NMDA [29] and slow GABA_A_[30, 31] receptor mediated currents are several tens of milliseconds. Afterdepolarizations have timescales of several tens of milliseconds as well [16, 32–35]. Another prominent class of slow inhibitory currents is mediated by GABA_B_ receptors and has time scales of one hundred to a few hundreds of milliseconds [36]. We remark that in our model the time constants of the afterdepolarization and the synaptic input currents may also be different without changing the dynamics: Assume that the synaptic time constant is different from that of the recovery current, but still satisfies the conditions that it is large against the inter-spike-intervals when the neuron is spiking and small against the timescale of [*x*_*n*_(*t*)]_+_. The synaptic current generated by the spike train of neuron *n* will then be approximately continuous and the filtering does not seriously affect its overall shape beyond smoothing out the spikes. As a consequence, the synaptic and the recovery currents are approximately proportional up to a constant factor that results from the different integrated contribution of individual spikes to them. Rescaling *γ* by this factor thus yields dynamics equivalent to the one with identical time constants.

#### Distributed encoding of continuous dynamics

In the above-described simple CSNs (CSNs with saturating synapses), each spiking neuron gives rise to one nonlinear continuous variable. The resulting condition that the inter-spike-intervals are small against the synaptic time constants if the neuron is spiking may in biological neural networks be satisfied for bursting or fast spiking neurons with slow synaptic currents. It will be invalid for different neurons and synaptic currents. The condition becomes unnecessary when the spiking neurons encode continuous variables collectively, i.e. if we partially replace the temporal averaging in *r*_*n*_(*t*) by an ensemble averaging. This can be realized by an extension of the above model, where only a lower, say *J*−, dimensional combination **x**(*t*) of the *N* − dimensional vectors **V**(*t*) and **r**(*t*) is continuous,

$$x\left(t\right)=LV\left(t\right)+\tilde{\Gamma }r\left(t\right),$$ (10)

where **L** and $\tilde{\Gamma }$ are *J*×*N* matrices (note that Eq (5) is a special case with *N* = *J* and diagonal matrices **L** and $\tilde{\Gamma }$). We find that spiking networks with nonlinear dendrites Eq (4) can encode such a lower dimensional variable **x**(*t*). The **x**(*t*) satisfy *J*-dimensional standard equations describing non-spiking continuous rate networks used for reservoir computing [2, 4, 26],

$$\dot{x}\left(t\right)=-\lambda_{x}x\left(t\right)+A\tanh\left(x(t)\right)+c\left(t\right).$$ (11)

We denote the resulting spiking networks as CSNs with nonlinear dendrites.

The derivation (see Supplementary material for details) generalizes the ideas introduced in refs. [12, 24, 37] to the approximation of nonlinear dynamical systems: We assume an approximate decoding equation (cf. also Eq (9)),

x(t)≈Γr(t), (12)

where **Γ** is a *J*×*N* decoding matrix and employ an optimization scheme that minimizes the decoding error resulting from Eq (12) at each time point. This yields the condition that a spike should be generated when a linear combination of **x**(*t*) and **r**(*t*) exceeds some constant value. We interpret this linear combination as membrane potential **V**(*t*). Solving for x(*t*) gives **L** and $\tilde{\Gamma }$ in terms of **Γ** in Eq (10). Taking the temporal derivative yields $\dot{V}\left(t\right)$, first in terms of $\dot{x}\left(t\right)$ and $\dot{r}\left(t\right)$ and after replacing them via Eqs (2),(11), in terms of **x**(*t*), **r**(*t*) and **s**(*t*). We then eliminate x(*t*) using Eq (12) and add a membrane potential leak term for biological realism and increased stability of numerical simulations. This yields Eq (4) together with the optimal values of the parameters given in Table 1. We note that the difference to the derivation in ref. [12] is the use of a nonlinear equation when replacing $\dot{x}\left(t\right)$. We further note that the spiking approximation of the continuous dynamics becomes exact, if in the last step **x**(*t*) is eliminated using Eq (10) and the leak term is omitted as it does not arise from the formalism in contrast to the case of CSNs with saturating synapses. Like in CSNs with saturating synapses, using the approximated decoding Eq (12) eliminates the biologically implausible **V**-dependencies in the interaction terms. For an illustration of this coding see Fig 1d.

### Learning universal computations

Recurrent continuous rate networks are a powerful means for learning of various kinds of computations, like steering of movements and processing of sequences [2, 4]. For this, an input and/or an output feedback signal needs to be able to “enslave” the network’s high-dimensional dynamics [27, 28]. This means that at any point in time the network’s state is a deterministic function of the recent history of input and feedback signals. The function needs to be high dimensional, nonlinear, and possess fading memory. A standard model generating suitable dynamics are continuous rate networks of the form Eq (11). Due to the typically assumed random recurrent connectivity, each neuron acts as a randomly chosen, nonlinear function with fading memory. Linearly combining them like basis functions by a linear readout can approximate arbitrary, nonlinear functions with fading memory (time-scales are limited by the memory of the network), and in this sense *universal computations* on the input and the feedback. The feedback can prolong the fading memory and allow to generate self-contained dynamical systems and output sequences [2–4, 38]. The feedback can be incorporated into the network by directly training the recurrent synaptic weights [4, 38].

**Fig 2:**
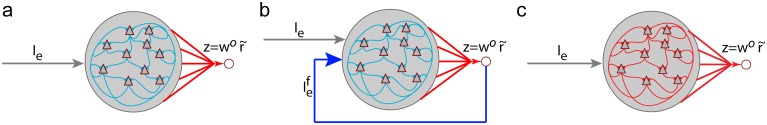
Setups used to learn versatile nonlinear computations with spiking neural networks. (a) A static *continuous signal coding spiking neural network* (*CSN*, gray shaded) serves as a spiking computational reservoir with high signal-to-noise ratio. The results of computations on current and past external inputs **I**_e_ can be extracted by simple neuron-like readouts. These linearly combine somatic inputs generated by saturating synapses or nonlinear dendrites, $\tilde{r}$ (red), to output signals **z** (Eqs (13, 14)). The output weights **w**^o^ are learned such that **z** approximates the desired continuous target signals. (b) *Plastic continuous signal coding spiking neural networks (PCSNs)* possess a loop that feeds the outputs **z** back via static connections as an additional input $I_{\text{e}}^{f}$ (blue, Eq (15)). Such networks have increased computational capabilities allowing them to, e.g., generate desired self-sustained activity. (c) The feedback loop can be incorporated into the recurrent network via plastic recurrent connections (red in gray shaded area).

Our understanding of the complex spiking dynamics of CSNs in terms of nonlinear first order differential equations enables us to apply the above theory to spiking neural networks: In the first step, we were able to conclude that our CSNs can generate enslaveable and thus computationally useful dynamics as they can be decoded to continuous dynamics that possess this property. In the second step, we have to ask which and how output signals should be learned to match a desired signal: In a biological setting, the appropriate signals are the sums of synaptic or dendritic input currents that spike trains generate, since these affect the somata of postsynaptic neurons as well as effectors such as muscles [39]. To perform, e.g., a desired continuous movement, they have to prescribe the appropriate muscle contraction strengths. For both CSNs with saturating synapses and with nonlinear dendrites, we choose the outputs to have the same form as the recurrent inputs that a soma of a neuron within the CSN receives. Accordingly, in our CSNs with saturating synapses, we interpret sums of the postsynaptic currents

$$z_{k}\left(t\right)=\sum_{m=1}^{N}w_{km}^{o}\tanh\left(\gamma r_{m}\left(t\right)\right)=:\sum_{m=1}^{N}w_{km}^{o}{\tilde{r}}_{m}\left(t\right)$$ (13)

as output signals, where the index *k* distinguishes *K*_out_ different outputs, and $w_{km}^{o}$ are the learnable synaptic output weights. For networks with nonlinear dendrites the outputs are a linear combination of inputs preprocessed by nonlinear dendrites

$$z_{k}\left(t\right)=\sum_{j=1}^{J}w_{kj}^{o}\tanh\left(\sum_{m=1}^{N}\Gamma_{jm}r_{m}\left(t\right)\right)=:\sum_{j=1}^{J}w_{kj}^{o}{\tilde{r}}_{j}\left(t\right),$$ (14)

where the strengths $w_{kj}^{o}$ of the dendro-somatic coupling are learned [40]. The networks can now learn the output weights such that *z*_*k*_(*t*) imitates a target signal *F*_*k*_(*t*), using standard learning rules for linear readouts (see Fig 2a for an illustration). We employ the recursive least squares method [41].

To increase the computational and learning abilities, the output signals should be fed back to the network as an (additional) input (Fig 2b)

$$I_{\text{e},\beta }^{f}\left(t\right)=\sum_{k=1}^{K_{\text{out}}}w_{\beta k}^{f}z_{k}\left(t\right)=\sum_{k=1}^{K_{\text{out}}}w_{\beta k}^{f}\sum_{\rho }w_{k\rho }^{o}{\tilde{r}}_{\rho }\left(t\right),$$ (15)

where each neuron receives a linear combination of the output signals *z*_*k*_(*t*) with static feedback connection strengths $w_{\beta k}^{f}$. Here and in the following Greek letter indices such as *β*,*ρ* range over all saturating synapses (*β*,*ρ* = 1,…,*N*; ${\tilde{r}}_{\beta }\left(t\right)=\tanh(\gamma r_{\beta }\left(t\right))$) in CSNs with saturating synapses, or over all nonlinear dendrites (*β*,*ρ* = 1,…,*J*; ${\tilde{r}}_{\beta }\left(t\right)=\tanh(\sum_{m=1}^{N}\Gamma_{\beta m}r_{m}\left(t\right))$) in CSNs with nonlinear dendrites.

It often seems biologically more plausible not to assume a strong feedback loop that enslaves the recurrent network, but rather to train recurrent weights. Our CSNs allow for this (Fig 2c): We can transform the learning of output weights in networks with feedback into mathematically equivalent learning of recurrent connection strengths, between synapses (CSNs with saturating synapses) or dendrites (CSNs with nonlinear dendrites) and the soma [40] (we learn *A*_*nm*_, see Methods for details of the implementation). We note that approximating different dynamical systems, e.g. ones equivalent to Eq (11) but with the coupling matrix inside the nonlinearity [42], may also in CSNs with nonlinear dendrites allow to learn synaptic weights in similar manner. We call CSNs with learning of outputs in presence of feedback, or with learning of recurrent connections *plastic continuous signal coding spiking neural networks (PCSNs).*

To learn feedback and recurrent connections, we use the FORCE imitation learning rule, which has recently been suggested for networks of continuous rate neurons [4, 38]: We use fast online learning based on the recursive least squares rule of the output weights in order to ensure that the output of the network is similar to the desired output at all times. Since during training the output is ensured to be close to the desired one, it can be used as feedback to the network at all times. The remaining deviations from the desired output are expected to be particularly suited as training noise as they reflect the system’s inherent noise. As mentioned before, the feedback loop may be incorporated in the recurrent network connectivity. During training, the reservoir connections are then learned in a similar manner as the readout.

In the following, we show that our approach allows spiking neural networks to perform a broad variety of tasks. In particular, we show learning of desired self-sustained dynamics at a degree of difficulty that has, to our knowledge, previously only been accessible with continuous rate networks.

### Applications

#### Self-sustained pattern generation

**Fig 3:**
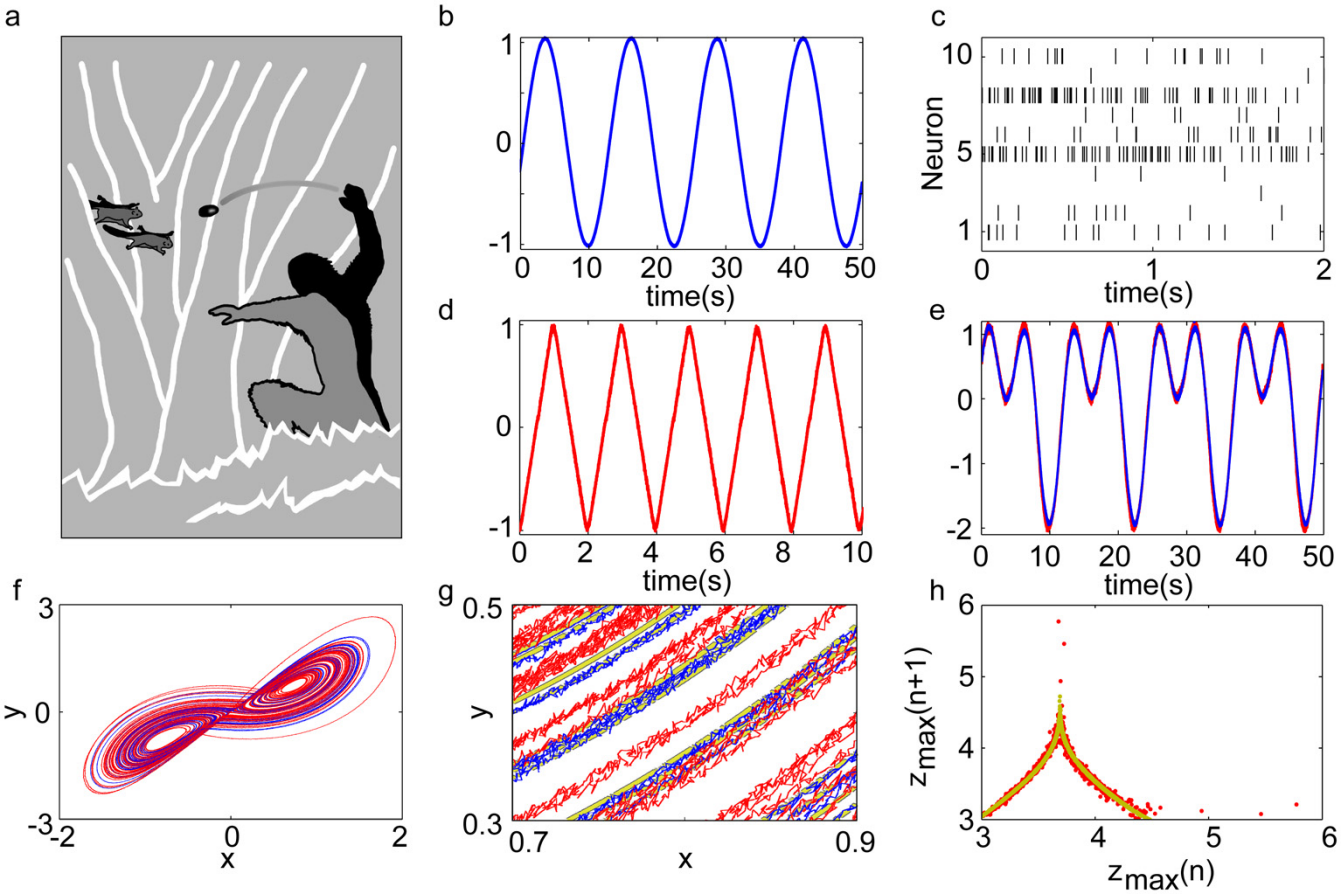
Learning dynamics with spiking neural networks. (a): Schematic hunting scene, illustrating the need for complicated dynamical systems learning and control. The hominid has to predict the motion of its prey, and to predict and control the movements of its body and the projectile. (b-h): Learning of self-sustained dynamical patterns by spiking neural networks. (b): A sine wave generated by summed, synaptically and dendritically filtered output spike trains of a PCSN with nonlinear dendrites. (c): A sample of the network’s spike trains generating the sine in (b). (d): A saw tooth pattern generated by a PCSN with saturating synapses. (e): A more complicated smooth pattern generated by both architectures (blue: nonlinear dendrites, red: saturating synapses). (f-h): Learning of chaotic dynamics (Lorenz system), with a PCSN with nonlinear dendrites. (f): The spiking network imitates an example trajectory of the Lorenz system during training (blue); it continues generating the dynamics during testing (red). (g): Detailed view of (f) highlighting how the example trajectory (yellow) is imitated during training and continued during testing. (h): The spiking network approximates not explicitly trained quantitative dynamical features, like the tent map between subsequent maxima of the z-coordinate. The ideal tent map (yellow) is closely approximated by the tent map generated by the PCSN (red). The spiking network sporadically generates errors, cf. the larger loop in (f) and the outlier points in (h). Panel (h) shows a ten times longer time series than (f), with three errors.

Animals including humans can learn a great variety of movements, from periodic patterns like gait or swimming, to much more complex ones like producing speech, generating chaotic locomotion [43, 44] or playing the piano. Moreover when an animal learns to use an object (Fig 3a), it has to learn the dynamical properties of the object as well as how its body behaves when interacting with it. Especially for complex, non-periodic dynamics, a dynamical system has to be learned with high precision.

How are spiking neural networks able to learn dynamical systems, store them and replay their activity? We find that PCSNs may solve the problem. They are able to learn periodic patterns of different degree of complexity as well as chaotic dynamical systems by imitation learning. Fig 3 illustrates this for PCSNs with nonlinear synapses (Fig 3d and 3e) and with nonlinear dendrites (Fig 3b, 3e and 3f).

The figure displays the recall of three periodic movements after learning: a sine wave, a more complicated non-differentiable saw tooth pattern and a “camel’s hump” superposition of sine and cosine. Also for long simulation times, we find no deviation from the displayed dynamics except for an inevitable phase shift (Fig Ga in S1 Text). It results from accumulation of small differences between the learned and desired periods. Apart from this, the error between the recalled and the desired signals is approximately constant over time (Fig Gb in S1 Text). This indicates that the network has learned a stable periodic orbit to generate the desired dynamics, the orbit is sufficiently stable to withstand the intrinsic noise of the system. Fig 3 furthermore illustrates learning of a chaotic dynamical system. Here, the network learns to generate the time varying dynamics of all three components of the Lorenz system and produces the characteristic attractor pattern after learning (Fig 3f). Due to the encoding of the dynamics in spike trains, the signal maintains a small deterministic error which emerges from the encoding of a continuous signal by discrete spikes (Fig 3g). The individual training and recall trajectories quickly depart from each other after the end of learning since they are chaotic. However, also for long simulation times, we observe qualitatively the same dynamics, indicating that the correct dynamical system was learned (Fig Gc in S1 Text). Occasionally, errors occur, cf. the larger loop in Fig 3f. This is to be expected due to the relatively short training period, during which only a part of the phase space covered by the attractor is visited. Importantly, we observe that after errors the dynamics return to the desired ones indicating that the general stability property of the attractor is captured by the learned system. To further test these observations, we considered a not explicitly trained long-term feature of the Lorenz-dynamics, namely the tent-map which relates the height *z*_*n* − 1_ of the (*n* − 1)th local maximum in the *z* − coordinate, to the height *z*_*n*_ of the subsequent local maximum. The spiking network indeed generates the map (Fig 3h), with two outlier points corresponding to each error.

In networks with saturating synapses, the spike trains are characterized by possibly intermittent periods of rather high-frequency spiking. In networks with nonlinear dendrites, the spike trains can have low frequencies and they are highly irregular (Fig 3c, Fig F in S1 Text). In agreement with experimental observations (e.g. [45]), the neurons can have preferred parts of the encoded signal in which they spike with increased rates.

The dynamics of the PCSNs and the generation of the desired signal are robust against dynamic and structural perturbations. They sustain noise inputs which would accumulate to several ten percent of the level of the threshold within the membrane time constant, for a neuron without further input (Fig B in S1 Text). For larger deviations of *W*_*njm*_ from their optimal values, PCSNs with nonlinear dendrites can keep their learning capabilities, if *μ* is tuned to a specific range. Outside this range, the capabilities break down at small deviations (Fig C in S1 Text). However, a slightly modified version of the models, where the reset is always to −*θ* (even if there was fast excitation that drove the neuron to spike by a suprathreshold input), has a high degree of robustness against such structural perturbations. We also checked that the fast connections are important, albeit substantial weakening can be tolerated (Fig D in S1 Text).

The deterministic spike code of our PCSNs encodes the output signal much more precisely than neurons generating a simple Poisson code, which facilitates learning. We have quantified this using a comparison between PCSNs with saturating synapses and networks of Poisson neurons of equal size, both learning the saw tooth pattern in the same manner. Since both codes become more precise with increasing spike rate of individual neurons, we compared the testing error between networks with equal spike rates. Due to their higher signal-to-noise ratio, firing rates required by the PCSNs to achieve the same pattern generation quality are more than one order of magnitude lower (Fig A in S1 Text).

#### Delayed reaction/time interval estimation

For many tasks, e.g. computations focusing on recent external input and generation of self-sustained patterns, it is essential that the memory of the involved recurrent networks is fading: If past states cannot be forgotten, they lead to different states in response to similar recent inputs. A readout that learns to extract computations on recent input will then quickly reach its capacity limit. In neural networks, fading memory originates on the one hand from the dynamics of single neurons, e.g. due to their finite synaptic and membrane time constants; on the other hand it is a consequence of the neurons’ connection to a network [46–48]. In standard spiking neural network models, the overall fading memory is short, of the order of hundreds of milliseconds [9–11, 49]. It is a matter of current debate how this can be extended by suitable single neuron properties and topology [1, 12, 50, 51]. Many biological computations, e.g. the simple understanding of a sentence, require longer memory, on the order of seconds.

**Fig 4:**
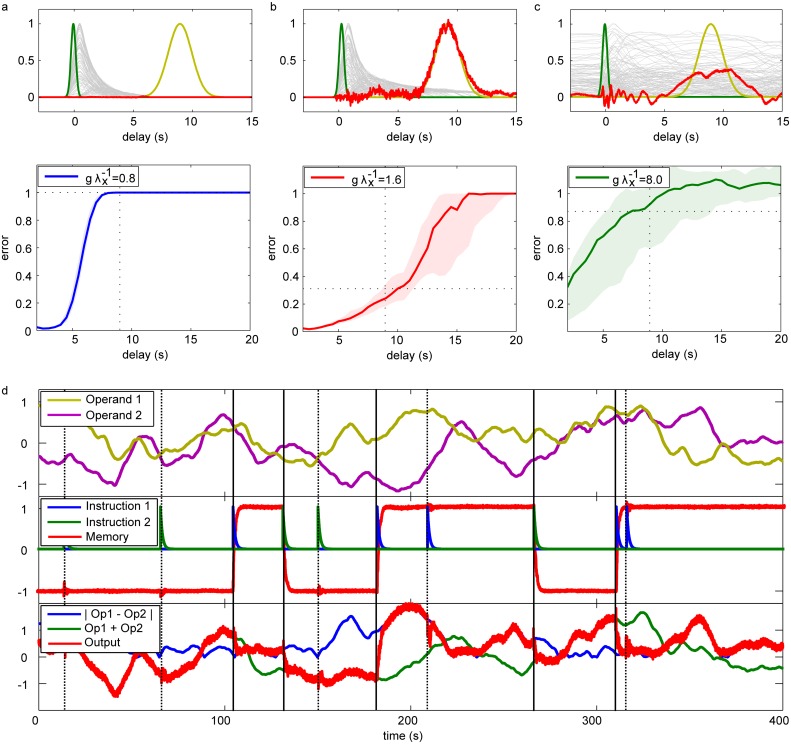
Learning of longer-term memory dependent computations with spiking neural networks. (a-c): Delayed reaction and time interval estimation: The synaptic output of a CSN learns to generate a generic reaction several seconds after a short input. Upper panels show typical examples of input, desired and actual reactions (green, yellow and red traces). In the three panels, the desired reaction delay is the same (9sec), the networks (CSNs with saturating synapses) have different levels of recurrent connection strengths ((a), (b), (c): low, intermediate, high level). The generation of the reaction is best for the network with intermediate level of connection strength. The CSNs with lower or higher levels have not maintained sufficient memory due to their extinguished or noisy and likely chaotic dynamics (gray background lines: spike rates of individual neurons). The median errors of responses measured for different delays in ensembles of networks (levels of connection strength as in the upper panels), are given in the lower panels. The shaded regions represent the area between the first and third quartile of the response errors. Dashed lines highlight delay and error size of the examples in the upper panels. (d): Persistent retaining of instructions and switching between computations: The network receives (i) two random continuous operand inputs (upper sub-panel, yellow and purple traces), and (ii) two pulsed instruction inputs (middle sub-panel, blue and green; memory of last instruction pulse: red). The network has learned to perform different computations on the operand inputs, depending on the last instruction (lower subpanel): if it was +1 (triggered by instruction channel 1), the network performs a nonlinear computation, it outputs the absolute value of the difference of the operands (red trace (network output) agrees with blue); if it was -1 (triggered by channel 2), the values of the operands are added (red trace agrees with green trace).

We find that CSNs without learning of recurrent connectivity or feedback access such time scales. We illustrate this by means of a delayed reaction/time estimation task: In the beginning of a trial, the network receives a short input pulse. By imitation learning, the network output learns to generate a desired delayed reaction. For this, it needs to specifically amplify the input’s dynamical trace in the recurrent spiking activity, at a certain time interval. The desired response is a Gaussian curve, representative for any type of delayed reaction. The reaction can be generated several seconds after the input (Fig 4a-4c).

The quality of the reaction pattern depends on the connection strengths within the network, specified by the spectral radius *g* of the coupling matrix divided by the leak of a single corresponding continuous unit λ_*x*_. Memory is kept best in an intermediate regime (Fig 4b), where the CSN stays active over long periods of time without overwriting information. This has also been observed for continuous rate networks [52]. For too weak connections (Fig 4a), the CSN returns to the inactive state after short time, rendering it impossible to retrieve input information later. If the connections are too strong, (Fig 4c), the CSN generates self-sustained, either irregular asynchronous or oscillating activity, partly overwriting information and hindering its retrieval. We observe that already the memory in disconnected CSNs with synaptic saturation can last for times beyond hundreds of milliseconds (cf. Fig E in S1 Text). This is a consequence of the recovery current: If a neuron has spiked several times in succession, the accumulated recovery current leads to further spiking (and further recovery current), and thus dampens the decay of a strong activation of the neuron [53].

Experiments show that during time estimation tasks, neurons are particularly active at two times: When the stimulus is received and when the estimated time has passed [54, 55]. Often the neuron populations that show activity at these points are disjoint. Our model reproduces this behavior for networks with good memory performance. In particular, at the time of the initial input the recurrently connected neurons become highly active (gray traces in Fig 4b, upper sub-panel) while at the estimated reaction time, readout neurons would show increased activity (red trace).

#### Persistent memory and context dependent switching

Tasks often also require to store memories persistently, e.g. to remember instructions [56]. Such memories may be maintained in learned attractor states (e.g. [57–60]). In the framework of our computing scheme, this requires the presence of output feedback [3]. Here, we illustrate the ability of PCSNs to learn and maintain persistent memories as attractor states as well as the ability to change behavior according to them. For this, we use a task that requires memorizing computational instructions (Fig 4d) [3]. The network has two types of inputs: After pulses in the instruction channels, it needs to switch persistently between different rules for computation on the current values of operand channels. To store persistent memory, the recurrent connections are trained such that an appropriate output can indicate the instruction channel that has sent the last pulse: The network learns to largely ignore the signal when a pulse arrives from the already remembered instruction channel, and to switch states otherwise. Due to the high signal-to-noise ratio of our deterministic spike code, the PCSNs are able to keep a very accurate representation of the currently valid instruction in their recurrent dynamics. Fig 4d, middle sub-panel, shows this by displaying the output of the linear readout trained to extract this instruction from the network dynamics. A similarly high precision can be observed for the output of the computational task, cf. Fig 4d, lower sub-panel.

#### Building of world models, and control

In order to control its environment, an animal has to learn the laws that govern the environment’s dynamics, and to develop a control strategy. Since environments are partly unpredictable and strategies are subject to evolutionary pressure, we expect that they may be described by stochastic optimal control theory. A particularly promising candidate framework is path integral control, since it computes the optimal control by simulating possible future scenarios under different random exploratory controls, and the optimal control is a simple weighted average of them [61]. For this, an animal needs an internal model of the system or tool it wants to act on. It can then mentally simulate different ways to deal with the system and compute an optimal one. Recent experiments indicate that animals indeed conduct thought experiments exploring and evaluating possible future actions and movement trajectories before performing one [62, 63].

**Fig 5:**
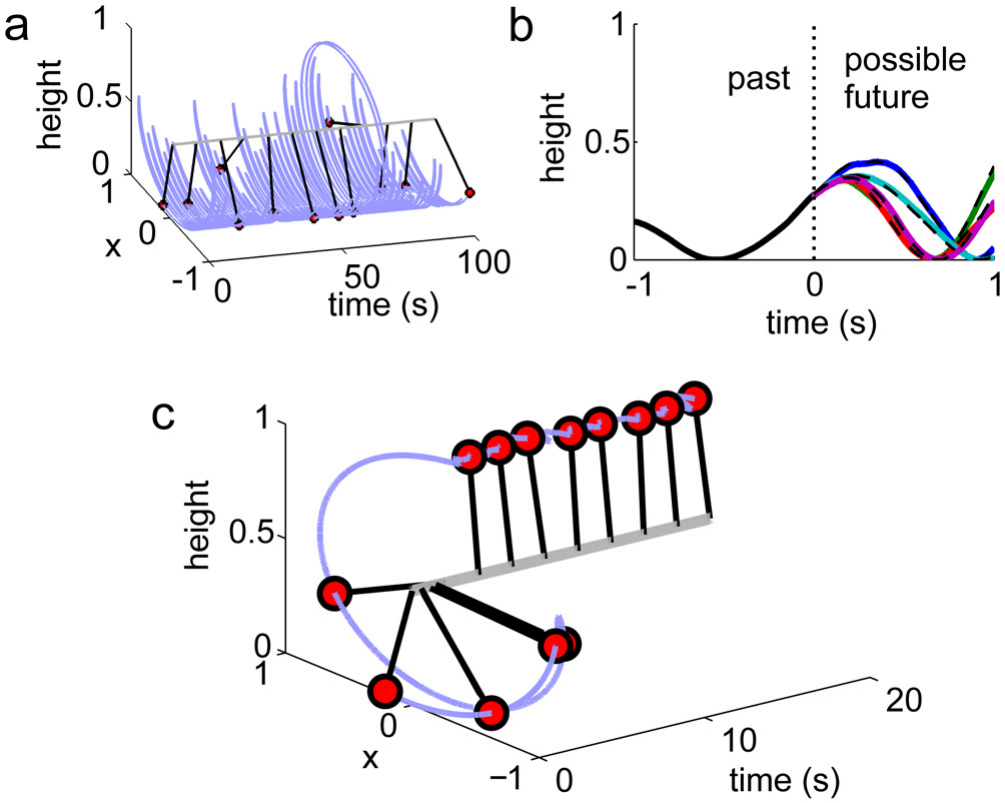
Model building and mental exploration to compute optimal control. (a): Learning of an internal world model with spiking neural networks. During model building, random exploratory control drives the dynamical system (here: a swinging pendulum). The spiking neural network is provided with the same control as input and learns to mimic the behavior of the pendulum as its output. (b): After learning, the spiking network can simulate the system’s response to control signals. The panel displays the height of the real pendulum in the past (solid black line) and future heights under different exploratory controls (dashed lines). For the same controls, the spiking neural network predicts very similar future positions (colored lines) as the imitated system. It can therefore be used for mental exploration and computation of optimal control to reach an aim, here: to invert the pendulum. (c): During mental exploration, the network simulates in regular time intervals a set of possible future trajectories for different controls, starting from the actual state of the pendulum. From this, the optimal control until the next exploration can be computed and applied to the pendulum. The control reaches its aim: The pendulum is swung up and held in inverted position, despite a high level of noise added during testing (uncontrolled dynamics as in panel (a)).

Here we show that by imitation learning, spiking neural networks, more precisely PCSNs with a feedback loop, can acquire an internal model of a dynamical system and that this can be used to compute optimal controls and actions. As a specific, representative task, we choose to learn and control a stochastic pendulum (Fig 5a and 5b). The pendulum’s dynamics are given by

$$\ddot{\phi }\left(t\right)+c\omega_{0}\dot{\phi }\left(t\right)+\omega_{0}^{2}\sin\left(\phi \left(t\right)\right)=\xi \left(t\right)+u\left(t\right),$$ (16)

with the angular displacement *ϕ* relative to the direction of gravitational acceleration, the undamped angular frequency for small amplitudes *ω*_0_, the damping ratio *c*, a random (white noise) angular force *ξ*(*t*) and the deterministic control angular force *u*(*t*), both applied to the pivot axis. The PCSN needs to learn the pendulum’s dynamics under largely arbitrary external control forces; this goes beyond the tasks of the previous sections. It is achieved during an initial learning phase characterized by motor babbling as observed in infants [64] and similarly in bird song learning [65]: During this phase, there is no deterministic control, *u* = 0, and the pendulum is driven by a random exploratory force *ξ* only. Also the PCSN receives *ξ* as input and learns to imitate the resulting pendulum’s dynamics with its output.

During the subsequent control phase starting at *t* = 0, the aim is to swing the pendulum up and hold it in the inverted position (Fig 5c). For this, the PCSN simulates at time *t* a set of *M* future trajectories of the pendulum, for different random exploratory forces *ξ*_*i*_ (“mental exploration” with *u* = 0, cf. Fig 5a and 5b), starting with the current state of the pendulum. In a biological system, the initialization may be achieved through sensory input taking advantage of the fact that an appropriately initialized output enslaves the network through the feedback. Experiments indicate that explored trajectories are evaluated, by brain regions separate from the ones storing the world model [66–68]. We thus assign to the simulated trajectories a reward *R*_*i*_ measuring the agreement of the predicted states with the desired ones. The optimal control *u*(*t* + *s*) (cf. Eq (16)) for a subsequent, not too large time interval *s* ∈ [0,*δ*] is then approximately given by a temporal average over the initial phase of the assumed random forces, weighted by the exponentiated total expected reward,

$$u(t+s)=\sum_{i=1}^{M}\frac{e^{\lambda_{c}R_{i}(t)}}{\sum_{j=1}^{M}e^{\lambda_{c}R_{j}(t)}}\bar{\xi_{i}}(t),$$ (17)

where ${\bar{\xi }}_{i}\left(t\right)={\frac{1}{\delta }}_{t}^{t+\delta }\xi_{i}\left(\tilde{t}\right)d\tilde{t}$ and λ_*c*_ is a weighting factor. We have chosen $R_{i}\left(t\right){=}_{t}^{t+T_{r}}y_{i}\left(\tilde{t}\right)d\tilde{t}$, i.e. the expected reward increases linearly with the heights $y_{i}\left(\tilde{t}\right)=-\cos\left(\phi_{i}\left(\tilde{t}\right)\right)$ predicted for the pendulum for input trajectory *ξ*_*i*_; it becomes maximal for a trajectory at the inversion point. *T*_*r*_ is the duration of a simulated trajectory. The optimal control is applied to the pendulum until *t* + Δ, with Δ < *δ*. Then, at *t* + Δ, the PCSN simulates a new set of trajectories starting with the pendulum’s updated state and a new optimal control is computed. This is valid and applied to the pendulum between *t* + Δ and *t* + 2Δ, and so on. We find that controlling the pendulum by this principle leads to the desired upswing and stabilization in the inversion point, even though we assume that the perturbing noise force *ξ* (Eq (16)) acting on the pendulum in addition to the deterministic control *u*, remains as strong as it was during the exploration/learning phase (cf. Fig 5a and 5b).

We find that for controlling the pendulum, the learned internal model of the system has to be very accurate. This implies that particular realizations of the PCSN can be unsuited to learn the model (we observed this for about half of the realizations), a phenomenon that has also been reported for small continuous rate networks before. However, we checked that continuous rate networks as encoded by our spiking ones reliably learn the task. Since the encoding quality increases with the number of spiking neurons, we expect that sufficiently large PCSNs reliably learn the task as well.

## Discussion

The characteristic means of communication between neurons in the nervous system are spikes. It is widely accepted that sequences of spikes form the basis of neural computations in higher animals. How computations are performed and learned is, however, largely unclear. Here we have derived *continuous signal coding spiking neural networks (CSNs),* a class of mesoscopic spiking neural networks that are a suitable substrate for computation. Together with plasticity rules for their output or recurrent connections, they are able to learn general, complicated computations by imitation learning (plastic CSNs, *PCSNs*). Learning can be highly reliable and accurate already for comparably small networks of hundreds of neurons. The underlying principle is that the networks reflect the input in a complicated nonlinear way, generate nonlinear transformations of it and use fading memory such that the inputs and their pasts interfere with each other. This requires an overall nonlinear relaxation dynamics suitable for computations [2]. Such dynamics are different from standard spiking neural network dynamics, which are characterized by a high level of noise and short intrinsic memory [9–11, 69].

To find spiking networks that generate appropriate dynamics, we use a linear decoding scheme for continuous signals encoded in the network dynamics as combinations of membrane potentials and synaptic currents. A specific coding scheme like this was introduced in refs. [12, 37] to derive spiking networks encoding linear dynamics in an optimal way. We introduce spiking networks where the encoded signals have dynamics desirable for computation, i.e. a nonlinear, high-dimensional, low-noise, relaxational character as well as significant fading memory. We conclude that, since we use simple linear decoding, already the dynamics of the spiking networks must possess these properties.

Using this approach, we study two types of CSNs: Networks with saturating synapses and networks with nonlinear dendrites. The CSNs with saturating synapses use a direct signal encoding; each neuron codes for one continuous variable. It requires spiking dynamics characterized by possibly intermittent phases of high rate spiking, or bursting, with inter-spike-intervals smaller than the synaptic time constants, which leads to a temporal averaging over spikes. Dynamics that appear externally similar to such dynamics were recently highlighted as a ‘second type of balanced state’ in networks of pulse-coupled, intrinsically oscillating model neurons [51]. Very recently [70, 71] showed that networks whose spiking dynamics are temporally averaged due to slow synapses possess a phase transition from a fixed point to chaotic dynamics in the firing rates, like the corresponding rate models that they directly encode. In the analytical computations the spike coding was not specified [70] or assumed to be Poissonian [71]. Numerical simulations of leaky integrate-and-fire neurons in the chaotic rate regime can generate intermittent phases of rather regular high-rate spiking [70]. The networks might provide a suitable substrate for learning computations as well. However, since the chaotic rate dynamics have correlations on the time scale of the slow synapses its applicability is limited to learning tasks where only a short fading memory of the reservoir is needed. For example delayed reaction tasks as illustrated in Fig 4a-4c would not be possible. Interestingly, in our scheme a standard leaky integrate-and-fire neuron with saturating synapses appears as a special case with recovery current of amplitude zero. According to our analysis it can act as a leaky integrator with a leak of the same time constant as the synapses, λ_*x*_ = λ_*s*_. In contrast, in presence of a recovery current, our networks with saturating synapses can encode slower dynamics on the order of seconds. After training the network, the time scales can be further extended.

In the CSNs with nonlinear dendrites the entire neural population codes for a usually smaller number of continuous variables, avoiding high firing rates in sufficiently large networks. The networks generate irregular, low frequency spiking and simultaneously a noise-reduced encoding of nonlinear dynamics, the temporal averaging over spikes in the direct coding case is partially replaced by a spatial averaging over spike trains from many neurons. The population coding scheme and our derivations of CSNs with nonlinear dendrites generalize the predictive coding proposed in ref. [12] to nonlinear dynamics. The roles of our slow and fast connections are similar to those used there: In particular, redundancies in the spiking are eliminated by fast recurrent connections without synaptic filtering. We expect that these couplings can be replaced by fast connections that have small finite synaptic time constants, as shown for the networks of ref. [12] in ref. [72]. In contrast to previous work, in the CSNs with nonlinear dendrites we have linear and nonlinear slow couplings. The former contribute to coding precision and implement linear parts of the encoded dynamics, the latter implement the nonlinearities in the encoded dynamics. Further, in contrast to previous work, the spike coding networks provide only the substrate for learning of general dynamical systems by adapting their recurrent connections. Importantly, this implies (i) that the neurons do not have to adapt their nonlinearities to each nonlinear dynamical system that is to be learned (which would not seem biologically plausible) and (ii) that the CSNs do not have to provide a faithful approximation of the nonlinear dynamics Eqs (6),(11), since a rough dynamical character (i.e. slow dynamics and the echo state property) is sufficient for serving as substrates. We note that refs. [73, 74] suggested to use the differential equations that characterize dynamical systems to engineer spiking neural networks that encode the dynamics. The approach suggests an alternative derivation of spiking networks that may be suitable as substrate for learning computations. Their rate coding scheme, however, allows for redundancy and thus higher noise levels, and it generates high frequency spiking. In a future publication, B. DePasquale, M. Churchland, and L.F. Abbott will present an approach to train rate coding spiking neural networks, with continuous rate networks providing the target signals [75]. We will discuss the relation between our and this approach in a joint review [76].

A characteristic feature of our neuron models is that they take into account nonlinearities in the synapses or in the dendrites. On the one hand this is biologically plausible [13, 19–21], on the other hand it is important for generating nonlinear computations. Our nonlinearities are such that the decoded continuous dynamics match those for typical networks of continuous rate neurons and provide a simple model for dendritic and synaptic saturation. However, the precise form of the neuron model and its nonlinearity is not important for our approaches: As long as the encoded dynamical system is suitable as a computational reservoir, the spiking system is a CSN and our learning schemes will work. As an example, a dendritic tree with multiple interacting compartments may be directly implemented in both the networks with saturating synapses and in the networks with nonlinear dendrites. A future task is to explore the computational capabilities of CSNs incorporating different and biologically more detailed features that lead to nonlinearities, e.g. neural refractory periods, dendritic trees with calcium and NMDA voltage dependent channels and/or standard types of short term synaptic plasticity.

Inspired by animals’ needs to generate and predict continuous dynamics such as their own body and external world movements, we let our networks learn to approximate desired continuous dynamics. Since effector organs such as muscles and post-synaptic neurons react to weighted, possibly dendritically processed sums of post-synaptic currents, we interpret these sums as the relevant, continuous signal-approximating outputs of the network [39]. Importantly, this is not the same as Poissonian rate coding of a continuous signal: As a simple example, consider a single spiking neuron. In our scheme it will spike with constant inter-spike-intervals to encode a constant output. In Poissonian rate coding, the inter-spike-intervals will be random, exponentially distributed and many more spikes need to be sampled to decode the constant output (cf. Fig A in S1 Text).

The outputs and recurrent connections of CSNs can be learned by standard learning rules [4, 41]. The weight changes depend on the product of the error and the synaptic or dendritic currents and may be interpreted as delta-rules with synapse- and time-dependent learning rates. PCSNs, with learning of recurrent weights or output feedback, show how spiking neural networks may learn internal models of complicated, self-sustained environmental dynamics. In our applications, we demonstrate that they can learn to generate and predict the dynamics in different depths, ranging from the learning of single stable patterns over the learning of chaotic dynamics to the learning of dynamics incorporating their reactions to external influences.

The spiking networks we use have medium size, like networks with continuous neurons used in the literature [2, 4]. CSNs with saturating synapses have, by construction, the same size as their non-spiking counterparts. In CSNs with nonlinear dendrites the spike load necessary to encode the continuous signals is distributed over the entire network. This leads to a trade-off between lower spiking frequency per neuron and larger network size (cf. Fig F in S1 Text): The faster the neurons can spike the smaller the network may be to solve a given task.

Previous work using spiking neurons as a reservoir to generate a high dimensional, nonlinear projection of a signal for computation, concentrated on networks without output feedback or equivalent task-specific learning of recurrent connectivity [1, 50, 77]. Such networks are commonly called “liquid state machines” [78]. By construction, they are unable to solve tasks like the generation of self-sustained activity and persistent memorizing of instructions; these require an effective output feedback, since the current output determines the desired future one: To compute the latter, the former must be made available to the network as an input. The implementation of spiking reservoir computers with feedback was hindered by the high level of noise in the relevant signals: The computations depend on the spike rate, the spike trains provide a too noisy approximation of this average signal and the noise is amplified in the feedback loop. While analytically considering feedback in networks of continuous rate neurons, ref. [3] showed examples of input-output tasks solved by spiking networks with a feedback circuit, the output signals are affected by a high level of noise. This concerns even output signals just keeping a constant value. We implemented similar tasks (Fig 4d), and find that our networks solve them very accurately due to their more efficient coding and the resulting comparably high signal-to-noise ratio. In contrast to previous work, our derivations systematically delineate spiking networks which are suitable for the computational principle with feedback or recurrent learning; the networks can accurately learn universal, complicated memory dependent computations as well as dynamical systems approximation, in particular the generation of self-sustained dynamics.

In the control task, we show how a spiking neural network can learn an internal model of a dynamical system, which subsequently allows to control the system. We use a path integral approach, which has already previously been suggested as a theory for motor control in biological systems [79, 80]. We apply it to learned world models, and to neural networks. Path integral control assumes that noise and control act in a similar way on the system [61]. This assumption is comparably weak and the path integral control method has been successfully applied in many robotics applications [81–83], where it was found to be superior to reinforcement learning and adaptive control methods.

Continuous rate networks using recurrence, readouts, and feedback or equivalent recurrent learning, are versatile, powerful devices for nonlinear computations. This has inspired their use in manifold applications in science and engineering, such as control, forecasting and pattern recognition [26]. Our study has demonstrated that it is possible to obtain similar performance using spiking neural networks. Therewith, our study makes spiking neural networks available for similarly diverse, complex computations and supports the feasibility of the considered computational principle as a principle for information processing in the brain.

## Methods

### Network simulation

We use a time grid based simulation scheme (step size *dt*). If not mentioned otherwise, between time points, we compute the membrane potentials using a Runge-Kutta integration scheme for dynamics without noise and an Euler-Maruyama integration scheme for dynamics with noise. Since CSNs with nonlinear dendrites have fast connections without conduction delays and synaptic filtering, we process spikings at a time point as follows: We test whether the neuron with the highest membrane potential is above threshold. If the outcome is positive, the neuron is reset and the impact of the spike on postsynaptic neurons is evaluated. Thereafter, we compute the neuron with the highest, possibly updated, membrane potential and repeat the procedure. If all neurons have subthreshold membrane potential, we proceed to the next time point. The described consecutive updating of neurons in a single time step increases in networks with nonlinear dendrites the robustness of the simulations against larger time steps, as the neurons maintain an order of spiking and responding like in a simulation with smaller time steps and a small but finite conduction delay and/or slight filtering of fast inputs. As an example, the scheme avoids that neurons that code for similar features and thus possess fast mutual inhibition, spike together within one step and generate an overshoot in the readout, as it would be the case in a parallel membrane potential updating scheme. The different tasks use either networks with saturating synapses or networks with nonlinear dendrites. In both cases, **A** is a sparse matrix with a fraction *p* of non-zero values. These are drawn independently from a Gaussian distribution with zero mean and variance $\frac{g^{2}}{pN}$ (CSNs with saturating synapses) or $\frac{g^{2}}{pJ}$ (CSNs with nonlinear dendrites), which sets the spectral radius of **A** approximately to *g*. For networks with nonlinear dendrites, the elements of **Γ** are drawn from a standard normal distribution. To keep the approach simple, we allow for positive and negative dendro-somatic couplings. In order to achieve a uniform distribution of spiking over the neurons in the network, we normalize the columns of **Γ** to have the same norm, which we control with the parameter *γ*_*s*_. This implies that the thresholds are identical.

### Training phase

The networks are trained for a period of length *T*_*t*_ such that the readouts *z*_*k*_ imitate target signals *F*_*k*_(*t*), i.e. such that the time average of the square of the errors *e*_*k*_(*t*) = *z*_*k*_(*t*) − *F*_*k*_(*t*) is minimized. At *T*_*t*_, training stops and the weights are not updated anymore in the subsequent testing. If present, the external input to the neurons is a weighted sum of *K*_in_ continuous input signals *f*_*k*_(*t*), $I_{\text{e,}\beta }\left(t\right)=\sum_{k=1}^{K_{\text{in}}}w_{\beta k}f_{k}\left(t\right)$, where the index *β* runs from 1 to *N* (CSNs with saturating synapses) or from 1 to *J* (CSNs with nonlinear dendrites). The weights *w*_*βk*_ are fixed and drawn from a uniform distribution in the range $\left[-{\tilde{w}}^{i},{\tilde{w}}^{i}\right]$. If present, the feedback weights $w_{\beta k}^{f}$ (cf. Eq (15)) are likewise chosen randomly from a uniform distribution in the range $\left[-{\tilde{w}}^{f},{\tilde{w}}^{f}\right]$ with a global feedback parameter ${\tilde{w}}^{f}$.

For the delayed reaction/time estimation task (Fig 4a-4c, Fig E in S1 Text), we applied the RLS (recursive least squares) algorithm [41] to learn the linear outputs. For the pattern generation, instruction switching and control tasks, we applied the FORCE (first-order reduced and controlled error) algorithm [4] (Figs 3, 4d and 5; Figs A-D, F and G in S1 Text) to learn the recurrent connections and linear outputs.

### Learning rules

The output weights $w_{km}^{o}$ are trained using the standard recursive least squares method [41]. They are initialized with 0, we use weight update intervals of Δ*t*. The weight update uses the current training error *e*_*k*_(*t*) = *z*_*k*_(*t*) − *F*_*k*_(*t*), where *z*_*k*_(*t*) is the output that should imitate the target signal *F*_*k*_(*t*), it further uses an estimate *P*_*βρ*_(*t*) of the inverse correlation matrix of the unweighted neural synaptic or dendritic inputs ${\tilde{r}}_{\beta }\left(t\right)$, as well as these inputs,

$$w_{k\beta }^{o}\left(t\right)=w_{k\beta }^{o}\left(t-\Delta t\right)-e_{k}\left(t\right)\sum_{\rho }P_{\beta \rho }\left(t\right){\tilde{r}}_{\rho }\left(t\right).$$ (18)

The indices *β*,*ρ* range over all saturating synapses (*β*,*ρ* = 1,…,*N*; ${\tilde{r}}_{\beta }\left(t\right)=\tanh(\gamma r_{\beta }\left(t\right))$) or all non-linear dendrites (*β*,*ρ* = 1,…,*J*; ${\tilde{r}}_{\beta }\left(t\right)=\tanh(\sum_{m=1}^{N}\Gamma_{\beta m}r_{m}\left(t\right))$) of the output neuron. The square matrix *P* is a running filter estimate of the inverse correlation matrix of the activity of the saturated synapses (CSNs with saturating synapses) or non-linear dendrites (CSNs with nonlinear dendrites). The matrix is updated via

$$P_{\beta \gamma }\left(t\right)=P_{\beta \gamma }\left(t-\Delta t\right)-\frac{\sum_{\rho }\sum_{\sigma }P_{\beta \rho }\left(t-\Delta t\right){\tilde{r}}_{\rho }\left(t\right){\tilde{r}}_{\sigma }\left(t\right)P_{\sigma \gamma }\left(t-\Delta t\right)}{1+\sum_{\rho }\sum_{\sigma }{\tilde{r}}_{\rho }\left(t\right)P_{\rho \sigma }\left(t-\Delta t\right){\tilde{r}}_{\sigma }\left(t\right)},$$ (19)

where the indices *β*,*γ*,*ρ*,*σ* run from 1 to *N* (CSNs with saturating synapses) or from 1 to *J* (CSNs with nonlinear dendrites). **P** is initialized as **P**(0) = *α*^−1^ **1** with *α*^−1^ acting as a learning rate.

For the update of output weights in presence of feedback and of recurrent weights we adopt the FORCE algorithm [4]. In presence of feedback, this means that recursive least squares learning of output is fast against the temporal evolution of the network, and already during training the output is fed back into the network. Thus, each neuron gets a feedback input

$$I_{\text{e},\beta }^{f}\left(t\right)=\sum_{k=1}^{K_{\text{out}}}w_{\beta k}^{f}z_{k}\left(t\right)=\sum_{k=1}^{K_{\text{out}}}w_{\beta k}^{f}\sum_{\rho }w_{k\rho }^{o}{\tilde{r}}_{\rho }\left(t\right).$$ (20)

The feedback weights $w_{\beta k}^{f}$ are static, the output weights are learned according to Eq (18).

Since the outputs are linear combinations of synaptic or dendritic currents, which also the neurons within the network linearly combine, the feedback loop can be implemented by modifying the recurrent connectivity, by adding a term $\sum_{k=1}^{K_{\text{out}}}w_{\rho k}^{f}w_{k\beta }^{o}$ to the matrix *A*_*ρβ*_. Learning then affects the output weights as well as the recurrent connections, separate feedback connections are not present. This learning and learning of output weights with a feedback loop are just two different interpretations of the same learning rule. For networks with saturating synapses the update is

$$A_{nm}\left(t\right)=A_{nm}\left(t-\Delta t\right)-\sum_{k=1}^{K_{\text{out}}}w_{nk}^{f}e_{k}\left(t\right)\sum_{l=1}^{N}P_{ml}\left(t\right){\tilde{r}}_{l}\left(t\right),$$ (21)

where the $w_{nk}^{f}$ are now acting as learning rates. For networks with nonlinear dendrites, the update is

$$D_{nj}\left(t\right)=D_{nj}\left(t-\Delta t\right)-\sum_{i=1}^{J}\Gamma_{in}\sum_{k=1}^{K_{\text{out}}}w_{ik}^{f}e_{k}\left(t\right)\sum_{h=1}^{J}P_{jh}\left(t\right){\tilde{r}}_{h}\left(t\right).$$ (22)

### Control task

The task is achieved in two phases, the learning and the control phase.

1. Learning: The PCSN learns a world model of the noisy pendulum, i.e. it learns the dynamical system and how it reacts to input. The pendulum follows the differential Eq (16) with *cω*_0_ = 0.1s^−1^ and $\omega_{0}^{2}=10{\text{s}}^{-2}$, *ξ*(*t*) is a white noise force with 〈*ξ*(*t*)*ξ*(*t*^′^)〉 = s^−3^ *δ*(*t* − *t*^′^), *x*(*t*) = sin(*ϕ*(*t*)) and *y*(*t*) = −cos(*ϕ*(*t*)) are Cartesian coordinates of the point mass. The neural network has one input and three outputs which are fed back into the network; it learns to output the *x*- and the *y*-coordinate, as well as the angular velocity of the pendulum when it receives as input the strength of the angular force (noise plus control) *ξ*(*t*) + *u*(*t*) applied to the pivot axis of the pendulum. The learning is here interpreted as learning in a network with feedback, cf. Eq (20).

We created a training trajectory of length *T*_*t*_ = 1000s by simulating the pendulum with the given parameters and by driving it with white noise *ξ*(*t*) as an exploratory control (*u*(*t*) = 0). Through its input, the PCSN receives the same white noise realization *ξ*(*t*). During training the PCSN learns to imitate the reaction of the pendulum to this control, more precisely its outputs learn to approximate the trajectories of *x*, *y* and *ω*. As feedback to the reservoir during training we choose a convex combination of the reservoir output and the target (feedback=0.9·output+0.1·target). We find that such a combination improves performance: If the output at the beginning of the training is very erroneous, those errors are accumulated through the feedback-loop, which prevents the algorithm from working. On the other hand, if one feeds back only the target signal, the algorithm does not learn how to correct for feedback transmitted readout errors. In our task, the convex combination alleviates both problems.

2. Control: In the second phase, the learned world model of the pendulum is used to compute stochastic optimal control that swings the pendulum up and keeps it in the inverted position. The PCSN does not learn its weights in this phase anymore. It receives the different realizations of exploratory (white noise) control and predicts the resulting motion (“mental exploration”). From this, the optimal control may be computed using the path integral framework [61]. In this framework a stochastic dynamical system (which is possibly multivariate)

$$\dot{x}\left(t\right)=f\left(x\left(t\right)\right)+u\left(x\left(t\right),t\right)+\xi \left(t\right)$$ (23)

with arbitrary nonlinearity **f**(**x**(*t*)) and white noise **ξ**(*t*), is controlled by the feedback controller **u**(**x**(*t*),*t*) to optimize an integral *C*(*t*) over a state cost $U(x\left(\tilde{t}\right))$ and a moving horizon quadratic control cost, $C\left(t\right){=}_{t}^{t+T_{r}}U\left(x\left(\tilde{t}\right)\right)+u{\left(\tilde{t}\right)}^{2}d\tilde{t}$. The reward is related to the cost by *R* = −*C*. Path integral control theory shows that the control at time *t* can be computed by generating samples from the dynamical system under the uncontrolled dynamics

$$\dot{x}\left(t\right)=f\left(x\left(t\right)\right)+\xi \left(t\right).$$ (24)

The control is then given by the success weighted average of the noise realizations *ξ*_*i*_

$$u\left(t\right)=\underset{\delta \to 0}{\text{lim}}\underset{M\to \infty }{\text{lim}}\overset{M}{\underset{i=1}{\sum }}\frac{e^{-\lambda_{c}C_{i}(t)}}{\sum_{j=1}^{M}e^{-\lambda_{c}C_{j}(t)}}\frac{1}{\delta }\int_{t}^{t+\delta }\xi_{i}\left(\tilde{t}\right)d\tilde{t},$$ (25)

where $C_{i}\left(t\right){=}_{t}^{t+T_{r}}U\left(x_{i}\left(\tilde{t}\right)\right)d\tilde{t}$ is the cost observed in the *i*th realization of the uncontrolled dynamics, which is driven by noise realization *ξ*_*i*_ and *u* = 0. Eq (17) is a discrete approximation to Eq (25). In our task, Eq (24) becomes

$$\begin{array}{c} \dot{\phi }\left(t\right)=\omega \left(t\right)\\ \dot{\omega }\left(t\right)=-\omega_{0}^{2}\sin\left(\phi \left(t\right)\right)-c\omega_{0}\omega \left(t\right)+\xi \left(t\right)+u\left(t\right) \end{array}$$

and *U*(**x**(*t*)) = −*y*(*t*) = cos(*ϕ*(*t*)).

### Figure details

The parameters of the different simulations are given in Table 2 for simulations using saturating synapses and in Table 3 for simulations using nonlinear dendrites. Further parameters and details about the figures and simulations are given in the following paragraphs.

**Table 2:** Parameters used in the different figures for simulations of networks with saturating synapses.

Sat. syn.	N	*α*	dt	*T*_*t*_	$\lambda_{s}^{-1}$	$\lambda_{V}^{-1}$	V_*r*_	*θ*
Fig 3d	50	0.1	0.1ms	100s	100ms	100ms	0.9*θ*	0.03
Fig 3e	50	0.1	1ms	100s	100ms	100ms	0.9*θ*	0.03
Fig 4a-4c	200	0.1	1ms	800s	100ms	50ms	0.54*θ*	0.1

**Table 3:** Parameters used in the different figures for simulations of networks with nonlinear dendrites. The parameter *a* = λ_*s*_ − λ_*x*_ is given in terms of λ_*s*_ and λ_*x*_.

Nonlin. dendr.	N	J	*α*	*γ*_*s*_	dt	*T*_*t*_	*μ*	$\lambda_{s}^{-1}$	$\lambda_{V}^{-1}$	a
Fig 3b,3c	500	50	0.1	0.03	1ms	100s	0	100ms	100ms	λ_*s*_ − 1s
Fig 3e	500	50	0.1	0.03	1ms	100s	0	100ms	100ms	λ_*s*_ − 1s
Fig 3f-3h	1600	800	0.1	0.03	1ms	200s	0	100ms	100ms	λ_*s*_ − 1s
Fig 4d	300	300	50	0.5	10ms	1000s	0	1s	0.5s	λ_*s*_ − 0.02s
Fig 5	500	300	0.1	0.03	1ms	1000s	20/*N*^2^	100ms	50ms	λ_*s*_ − 10s

If not mentioned otherwise, for all simulations we use $g=1.5\frac{1}{\text{s}}$, *p* = 0.1, ${\tilde{w}}^{f}=1\frac{1}{\text{s}}$, ${\tilde{w}}^{i}=1\frac{1}{\text{s}}$, Δ*t* = 0.01s, *γ* = *θ* and $\sigma_{\eta }=0\frac{1}{\sqrt{\text{s}}}$. We note that for simulations with saturating synapses, we model the slow synaptic currents to possess synaptic time constants of 100ms (cf., e.g., [50, 60]). We usually use the same value for the slow synapses in networks with nonlinear dendrites. Upon rescaling time, these networks can be interpreted as networks with faster time constants, which learn faster target dynamics. Since the spike rates scale likewise, we have to consider larger networks to generate rates in the biologically plausible range (cf. Fig F in S1 Text).

#### Figure 3

Figure 3b, 3c: The PCSN has non-linear dendrites. The target signal is a sine with period 4*π*s and amplitude 2 (normalized to one in the figure). During recall, the neurons of the PCSN spike with mean rate 30.2Hz.

Figure 3d: The PCSN has saturating synapses. The target signal is a saw tooth pattern with period 2s and amplitude 10 (normalized to one in the figure). We used an Euler scheme here. The mean spike rate is 226Hz.

Figure 3e: The task is performed by a PCSN with non-linear dendrites and by a PCSN with saturating synapses. The target signal is $\sin\left(t\frac{0.5}{\text{s}}\right)+\cos\left(t\frac{1}{\text{s}}\right)$. The mean spike rate is 77.8Hz for saturating synapses and 21.3Hz for non-linear dendrites.

Figure 3f-3h: The PCSN has nonlinear dendrites. As teacher we use the standard Lorenz system

$$\begin{array}{c} \dot{x}\left(t\right)=\sigma \left(y\left(t\right)-x\left(t\right)\right)\\ \dot{y}\left(t\right)=x\left(t\right)\left(\rho -z\left(t\right)\right)-y\left(t\right)\\ \dot{z}\left(t\right)=x\left(t\right)y\left(t\right)-\beta z\left(t\right) \end{array}$$

with parameters *σ* = 10, *ρ* = 28, *β* = 8/3; we set the dimensionless temporal unit to 0.2s and scale the dynamical variables by a factor of 0.1. Panels (f,g) show a recall phase of 400s, panel (h) shows points from a simulation of 4000s. Panel (f) only shows every 10th data point, panel (g) shows every data point. The mean spike rate is 432Hz.

#### Figure 4

Figure 4a-4c: We quantified the memory capacity of a CSN with saturating synapses. The network has a sparse connectivity matrix **A** without autapses. We applied white noise with $\sigma_{\eta }=0.001\frac{1}{\sqrt{\text{s}}}$. The input is a Gaussian bell curve with *σ* = 0.2s and integral 10s (height normalized to one in the figure). The target is a Gaussian bell curve with *σ* = 1s and integral 1s (height normalized to one in the figure). The target is presented several seconds after the input. Trials consisting of inputs and subsequent desired outputs are generated at random times with exponential inter-trial-interval distribution with time constant 10s and a refractory time of 100s. Training time is *T*_*t*_ = 800s, i.e. the network is trained with about 6 to 8 trials. Testing has the same duration with a similar number of trials. There is no feedback introduced by initialization or by learning, so the memory effect is purely inherent to the random network. We compute the quality of the desired output generation as the root mean squared (RMS) error between the generated and the desired response, normalized by the number of test trials. As reference, we set the error of the “extinguished” network, which does not generate any reaction to the input, to 1. Lower panels of Fig 4a-4c display medians and quartiles taken over 50 task repetitions. The sweep was done for time-delays 2 − 20s in steps of 0.5 s.

Figure 4d: The PCSN has nonlinear dendrites. For this task a constant input of $I_{e}^{\text{const}}=b$ is added to the network with the elements of the vector **b** chosen uniformly from $\left[0\frac{1}{\text{s}},250\frac{1}{\text{s}}\right]$ to introduce inhomogeneity. Four different inputs are fed into the network, two continuous $f_{1/2}^{c}$ and two pulsed input channels $f_{1/2}^{p}$. The continuous inputs are created by convolving white noise twice with an exponential kernel $e^{-t\frac{1}{\text{s}}}$ (equivalent to convolving once with an alpha function) during training and $e^{-t\frac{1}{10\text{s}}}$ during testing. The continuous input signals are normalized to have mean 0 and standard deviation 0.5. The pulsed instruction input is created by the convolution of a Poisson spike train with an exponential kernel $e^{-t\frac{1}{\text{s}}}$. The rate of the delta pulses during training is $0.04\frac{1}{\text{s}}$. During testing we choose a slower rate of $0.01\frac{1}{\text{s}}$ for a clearer presentation. In the rare case when two pulses overlap such that the pulsed signal exceeds an absolute value of 1.01 times the maximal pulse height of one, we shift the pulse by the minimal required amount of time to achieve a sum of the pulses below or equal to 1.01. We use weights ${\tilde{w}}^{i,p}=100\frac{1}{\text{s}}$ for the pulsed inputs, ${\tilde{w}}^{i,c}=250\frac{1}{\text{s}}$ for the continuous inputs and ${\tilde{w}}^{f}=250\frac{1}{\text{s}}$ for the feedback; $g=75\frac{1}{\text{s}}$. The recurrent weights of the network are trained with respect to the memory target *F*_*m*_(*t*). This target is +1 if the last instruction pulse came from $f_{1}^{p}$ and it is −1 if the last pulse came from $f_{2}^{p}$. During switching the target follows the integral of the input pulse. The corresponding readout is *z*_*m*_. The second readout *z*_*c*_ is trained to output the absolute value of the difference of the two continuous inputs, if the last instruction pulse came from $f_{1}^{p}$, and to output their sum, if the last instruction pulse came from $f_{2}^{p}$. The specific analytical form of this target is $F_{c}\left(t\right)=|f_{1}^{c}\left(t\right)-f_{2}^{c}\left(t\right)|\left(F_{m}\left(t\right)+1\right)/2-\left(f_{1}^{c}\left(t\right)+f_{2}^{c}\left(t\right)\right)\left(F_{m}\left(t\right)-1\right)/2$. The mean spike rate is 5.53Hz.

#### Figure 5

Since we have white noise as input we use the Euler-Maruyama scheme in all differential equations. The PCSN has nonlinear dendrites. Non-plastic coupling strengths are ${\tilde{w}}^{f,y}=100\frac{1}{\text{s}}$ for the feedback of the y-coordinate, ${\tilde{w}}^{f,x}=100\frac{1}{\text{s}}$ for the feedback of the x-coordinate, ${\tilde{w}}^{f,\omega }=20\frac{1}{\text{s}}$ for the feedback of the angular velocity and ${\tilde{w}}^{i}=\frac{2}{7}\frac{1}{\text{s}}$ for the input. We introduce an additional random constant bias term into the nonlinearity to increase inhomogeneity between the neurons: The nonlinearity is $\tanh(b_{j}+\sum_{m=1}^{N}W_{njm}r_{m}\left(t\right))$ where *b*_*j*_ is drawn from a Gaussian distribution with standard deviation 0.01. The integration time *δ* is 0.1s. During the control/testing phase, every Δ = 0.01s, *M* = 200 samples of length *T*_*r*_ = 1s are created, the cost function is weighted with $\lambda_{c}=0.01\frac{1}{\text{s}}$. The mean spike rate is 146Hz.

## Supporting Information

S1 Text Supporting text, figures and methods. (PDF)
