# Supplementary material for: Learning Universal Computations with Spikes
Source: PLoS Comput Biol. 2016 Jun 16;12(6):e1004895. doi: 10.1371/journal.pcbi.1004895 (PMC4911146; doi:10.1371/journal.pcbi.1004895)
Supplement: S1 Text — (PDF) [file pcbi.1004895.s001.pdf]

Learning universal computations with spikes - S1 Supporting information

Dominik Thalmeier, Marvin Uhlmann, Hilbert J. Kappen, and Raoul-Martin Memmesheimer

1 Supporting text and figures

1.1 Networks with nonlinear dendrites

As stated in the main text, we can generalize Equation (3) by introducing fast connections that generate discontinuities in postsynaptic neurons when a neuron spikes. We may then require that only a lower, say $J-$, dimensional combination $\mathbf{x}(t)$ of the $N-$dimensional vectors $\mathbf{V}(t)$ and $\mathbf{r}(t)$ is continuous,

$$\mathbf{x}(t) = \mathbf{L}\mathbf{V}(t) + \tilde{\mathbf{\Gamma}}\mathbf{r}(t), \tag{S1}$$

where $\mathbf{L}$ and $\tilde{\mathbf{\Gamma}}$ are $J \times N$ matrices. (For clarity of presentation we will use vector/matrix notation instead of components throughout the present section.) The benefit of this approach is that the spike trains of a larger population of neurons contribute to each x_n, such that a modified analogue to Equation (9),

$$\mathbf{x}(t) \approx \mathbf{\Gamma}\mathbf{r}(t), \tag{S2}$$

with a $J \times N$ matrix $\mathbf{\Gamma}$ can hold even if the spike rates of individual neurons are low, i.e. if we make use of population/distributed coding. The matrix $\mathbf{L}$ is fixed (except for degenerate cases) as soon as the matrix $\tilde{\mathbf{\Gamma}}$ and the fast changes in $\mathbf{V}$ are fixed. However, it is not a priori clear how to choose the latter two; we need to employ some optimization scheme to ensure both a good approximation Equation (S2) and a low firing rate.

For this, we start anew, and in contrast to the previous section with the dynamics for $\mathbf{x}(t)$. From these we will derive spiking dynamics approximating the $\mathbf{x}(t)$. We begin with a general J-dimensional nonlinear dynamical system yielding $\mathbf{x}(t)$,

$$\dot{\mathbf{x}}(t) = \mathbf{f}(\mathbf{x}(t)) + \mathbf{c}(t), \tag{S3}$$

where $\mathbf{f}(\mathbf{x})$ and $\mathbf{c}(t)$ are column vectors of functions $f_j(x_1, ..., x_N)$ and external inputs $c_j(t)$, respectively. We will generalize an approach introduced in refs. [1, 2, 3] to nonlinear systems and derive spiking dynamics that optimally (see below) approximate $\mathbf{x}(t)$ satisfying Equation (S3). The approach will yield Equation (S1) with a specific $\mathbf{L}$ as by-product. We will find that the dynamics of individual neurons depend on the f_j and we will specify these functions such that the neural dynamics are biologically plausible and suitable for universal computation.

We choose the momentary error or cost function

$$E(t) = (\mathbf{x}(t) - \mathbf{\Gamma}\mathbf{r}(t))^2 + \mu\mathbf{r}^2(t) \tag{S4}$$

to be minimized at each time t. The first term in $E(t)$ induces the approximation Equation (S2), the second term fosters a low spike rate with spiking distributed over all neurons. The error function respects causality as it depends implicitly via $\mathbf{x}(t)$ and $\mathbf{r}(t)$ on the past and restrains the dynamics at the current time t only. Minimizing $E(t)$ at t means that a spike should be sent by neuron n if $E(t)$ decreases due to this spike. Comparing $E_n(t)$ (spike sending at time t by neuron n) with $E_0(t)$ (no spike sending) yields

$$E_n(t) < E_0(t) \tag{S5}$$

$$(\mathbf{x}(t) - \mathbf{\Gamma r}(t) - \mathbf{\Gamma}\hat{\mathbf{e}}_n)^2 + \mu\left(\mathbf{r}(t) + \hat{\mathbf{e}}_n\right)^2 < (\mathbf{x}(t) - \mathbf{\Gamma r}(t))^2 + \mu\mathbf{r}^2(t) \tag{S6}$$

$$\mathbf{\Gamma}_n \cdot (\mathbf{x}(t) - \mathbf{\Gamma r}(t)) - \mu r_n(t) > \frac{\mathbf{\Gamma}_n^2 + \mu}{2}, \tag{S7}$$

where $\hat{\mathbf{e}}_n$ denotes the n-th unit vector, $\hat{\mathbf{e}}_n = (0, ..., 1, 0, ...)^T$ (with a 1 in the n-th row), and $\mathbf{\Gamma}_n$ is the n-th column (vector) of the matrix $\mathbf{\Gamma}$, $\mathbf{\Gamma}_n = \mathbf{\Gamma}\hat{\mathbf{e}}_n$. To obtain the familiar condition $V_n(t) > \theta_n$ for neuron n to spike, the variable left hand side of Equation (S7) may be interpreted as membrane potential,

$$V_n(t) = \mathbf{\Gamma}_n \cdot (\mathbf{x}(t) - \mathbf{\Gamma r}(t)) - \mu r_n(t), \tag{S8}$$

$$\mathbf{V}(t) = \mathbf{\Gamma}^T\left(\mathbf{x}(t) - \mathbf{\Gamma r}(t)\right) - \mu\mathbf{r}(t), \tag{S9}$$

the right hand side as threshold

$$\theta_n = \frac{\mathbf{\Gamma}_n^2 + \mu}{2}. \tag{S10}$$

We note that we can multiply both sides of the Equation by a factor and add constant terms, these change the scale of the potential, and shift the resting membrane potential, the reset and the threshold. Equation (S9) yields Equation (S1) with the pseudo-inverse of $\mathbf{\Gamma}^T$, $\mathbf{L} = (\mathbf{\Gamma\Gamma}^T)^{-1}\mathbf{\Gamma}$, and $\tilde{\mathbf{\Gamma}} = \mathbf{\Gamma} + \mu\mathbf{L}$.

We can now derive the sub-threshold dynamical Equations for $\mathbf{V}(t)$ from those for $\mathbf{x}(t)$ and $\mathbf{r}(t)$:

$$\dot{\mathbf{V}}(t) = \mathbf{\Gamma}^T\left(\dot{\mathbf{x}}(t) - \mathbf{\Gamma}\dot{\mathbf{r}}(t)\right) - \mu\dot{\mathbf{r}}(t) \tag{S11}$$

$$= \mathbf{\Gamma}^T\mathbf{f}(\mathbf{x}(t)) - \left(\mathbf{\Gamma}^T\mathbf{\Gamma} + \mu\mathbf{1}\right)(\mathbf{s}(t) - \lambda_s\mathbf{r}(t)) + \mathbf{\Gamma}^T\mathbf{c}(t), \tag{S12}$$

where $\mathbf{1}$ denotes the $N \times N$ identity matrix. Assuming that the minimization of Equation (S4) yields small $E(t)$, we may eliminate the dependence on $\mathbf{x}(t)$ using Equation (S2),

$$\dot{\mathbf{V}}(t) \approx \mathbf{\Gamma}^T\mathbf{f}(\mathbf{\Gamma r}(t)) - \left(\mathbf{\Gamma}^T\mathbf{\Gamma} + \mu\mathbf{1}\right)(\mathbf{s}(t) - \lambda_s\mathbf{r}(t)) + \mathbf{\Gamma}^T\mathbf{c}(t). \tag{S13}$$

Finally, biological realism and increased stability of numerical simulations indicate that an additional leak term $-\lambda_V\mathbf{V}$ should be introduced

$$\dot{\mathbf{V}}(t) = -\lambda_V\mathbf{V}(t) + \mathbf{\Gamma}^T\mathbf{f}(\mathbf{\Gamma r}(t)) - \left(\mathbf{\Gamma}^T\mathbf{\Gamma} + \mu\mathbf{1}\right)(\mathbf{s}(t) - \lambda_s\mathbf{r}(t)) + \mathbf{\Gamma}^T\mathbf{c}(t). \tag{S14}$$

We now choose the f_j as

$$f_j(x_1, ..., x_J) = -\lambda_x x_j + \sum_{i=1}^{J} A_{ji}\tanh(x_i), \tag{S15}$$

such that

$$\dot{\mathbf{V}}(t) = -\lambda_V \mathbf{V}(t) + \mathbf{\Gamma}^T \mathbf{A} \tanh(\mathbf{\Gamma}\mathbf{r}(t)) - \left(\mathbf{\Gamma}^T\mathbf{\Gamma} + \mu \mathbf{1}\right) \mathbf{s}(t) + \left(a\mathbf{\Gamma}^T\mathbf{\Gamma} + \mu\lambda_s \mathbf{1}\right) \mathbf{r}(t) + \mathbf{\Gamma}^T \mathbf{c}(t), \quad \text{(S16)}$$

where $a = \lambda_s - \lambda_x$. This yields a spiking neural network that is suitable for universal computation: Its dynamics can be decoded via Equation (S1) (or Equation (S2)) to resemble those of a J-dimensional dynamical system of the form

$$\dot{\mathbf{x}}(t) = -\lambda_x \mathbf{x}(t) + \mathbf{A} \tanh\left(\mathbf{x}(t)\right) + \mathbf{c}(t). \quad \text{(S17)}$$

Systems of the form Equation (S17) are known to be suitable for universal computation [4, 5, 6], in particular for appropriate $\mathbf{A}$ they can maintain longer-term fading memory. Since the $\mathbf{x}$ are dynamical quantities linearly derived from the underlying spiking network, already the underlying spiking network is suitable for computations.

Furthermore, the structure of Equation (S16) allows for a straightforward interpretation in biological terms: The response of neuron n's soma to slow input to its J dendrites is modeled by the term $\mathbf{\Gamma}_n \cdot (\mathbf{A} \tanh(\mathbf{\Gamma}\mathbf{r}(t)))$. The inputs have non-negligible synaptic time constant (cf. $\mathbf{r}(t)$), they are linearly summed and thereafter subjected to a dendritic sublinearity ($\tanh$). The coupling strength of a synaptic connection from neuron m to the jth dendrite of neuron n is given by Γ_{jm}, the coupling strength from the jth dendrite of neuron n to its soma is $(\mathbf{\Gamma}^T\mathbf{A})_{nj}$. Further fast and slow inputs arriving near the soma (and thus not subject to a dendritic non-linearity) are incorporated by the terms $-\left(\mathbf{\Gamma}^T\mathbf{\Gamma} + \mu\mathbf{1}\right)\mathbf{s}(t)$ and $\left(a\mathbf{\Gamma}^T\mathbf{\Gamma} + \mu\lambda_s\mathbf{1}\right)\mathbf{r}(t)$. Their impact is characterized by the product $\mathbf{\Gamma}^T\mathbf{\Gamma}$ of the decoding matrix with itself and the comparably small weight μ of the spike frequency penalty term, the positive diagonal terms incorporate the reset of the neurons after a spike and a slower recovery.

1.2 A sufficient condition for the echo state property of the dynamics Equation (6)

When does

$$\dot{\mathbf{x}}(t) = -\lambda_V \left[\mathbf{x}(t)\right]_- - \lambda_x \left[\mathbf{x}(t)\right]_+ + \mathbf{A} \tanh(\left[\mathbf{x}(t)\right]_+) + I(t) \quad \text{(S18)}$$

(Equation (6) of the main text) possess the echo state property? Dynamics have this property if after sufficiently long time any initial conditions are washed out and the state of the system is completely determined by the input. This is definitely the case if the distance between trajectories decays at least exponentially with a rate independent of the input [7]. We will prove the latter for our dynamics Equation (S18). For this, we will consider the difference $\mathbf{\Delta}(t) = \mathbf{x}_1(t) - \mathbf{x}_2(t)$ and the Euclidean distance $\|\mathbf{\Delta}(t)\|$ of two trajectories that satisfy Equation (S18) and have different initial conditions $\mathbf{x}_1(0)$,$\mathbf{x}_2(0)$ but the same input $I(t)$. We will show that under the condition $\|\mathbf{A}\| < \min(\lambda_V, \lambda_x)$, with $\|\mathbf{A}\|$ being the spectral norm (the largest singular value) of $\mathbf{A}$, an inequality $\dot{\|\mathbf{\Delta}(t)\|} \leq -\epsilon \|\mathbf{\Delta}(t)\|$ holds for some $\epsilon > 0$ (as usual the dot denotes the temporal derivative of the entire expression below, here $\|\mathbf{\Delta}(t)\|$).

We start with the expression $\frac{1}{2}\dot{\|\mathbf{\Delta}(t)\|^2} = \mathbf{\Delta}(t)\dot{\mathbf{\Delta}}(t)$ and replace the right hand side using $\mathbf{\Delta}(t) =$

$\mathbf{x}_1(t) - \mathbf{x}_2(t)$ and Equation (S18), which leads to

$$\begin{aligned}\frac{1}{2}\dot{\|\boldsymbol{\Delta}(t)\|^2} &= (\mathbf{x}_1 - \mathbf{x}_2)\left(-\lambda_V\left([\mathbf{x}_1(t)]_- - [\mathbf{x}_2(t)]_-\right) - \lambda_x\left([\mathbf{x}_1(t)]_+ - [\mathbf{x}_2(t)]_+\right)\right) \\ &\quad + (\mathbf{x}_1 - \mathbf{x}_2)\left(\mathbf{A}\left(\tanh([\mathbf{x}_1(t)]_+) - \tanh([\mathbf{x}_2(t)]_+)\right) + I(t) - I(t)\right). \qquad \text{(S19)}\end{aligned}$$

To proceed we use the three inequalities $\mathbf{x}\mathbf{y} \leq \|\mathbf{x}\|\,\|\mathbf{y}\|$, $\|\mathbf{A}\mathbf{x}\| \leq \|\mathbf{A}\|\,\|\mathbf{x}\|$ and $\|\tanh(\mathbf{x}) - \tanh(\mathbf{y})\| \leq \|\mathbf{x} - \mathbf{y}\|$, which allow to estimate

$$(\mathbf{x}_1 - \mathbf{x}_2)\,\mathbf{A}\left(\tanh([\mathbf{x}_1(t)]_+) - \tanh([\mathbf{x}_2(t)]_+)\right) \leq \|\mathbf{x}_1 - \mathbf{x}_2\|\,\|\mathbf{A}\|\,\left\|[\mathbf{x}_1(t)]_+ - [\mathbf{x}_2(t)]_+\right\|. \qquad \text{(S20)}$$

We now simplify the right hand side of the inequality further. For this we use that for every pair of real valued vectors $\mathbf{x}$ and $\mathbf{y}$ we have $[\mathbf{x}]_\pm [\mathbf{y}]_\pm \geq 0$, $[\mathbf{x}]_\pm [\mathbf{y}]_\mp \leq 0$ and $[\mathbf{x}]_\pm [\mathbf{x}]_\mp = 0$, since every element of $[\mathbf{x}]_+$ is larger/equal zero while every element of $[\mathbf{x}]_-$ is smaller/equal zero, and elements which are nonzero in $[\mathbf{x}]_+$ are zero in $[\mathbf{x}]_-$ and vice versa. With this we get

$$\begin{aligned}\|\mathbf{x}_1 - \mathbf{x}_2\|^2 &= \left\|[\mathbf{x}_1(t)]_+ - [\mathbf{x}_2(t)]_+\right\|^2 + \left\|[\mathbf{x}_1(t)]_- - [\mathbf{x}_2(t)]_-\right\|^2 \\ &\quad -2\,[\mathbf{x}_1(t)]_+\,[\mathbf{x}_2(t)]_- - 2\,[\mathbf{x}_2(t)]_+\,[\mathbf{x}_1(t)]_- \\ &\geq \left\|[\mathbf{x}_1(t)]_+ - [\mathbf{x}_2(t)]_+\right\|^2, \qquad \text{(S21)}\end{aligned}$$

since $-\,[\mathbf{x}_1(t)]_+\,[\mathbf{x}_2(t)]_- - [\mathbf{x}_2(t)]_+\,[\mathbf{x}_1(t)]_- \geq 0$. The result can be used to bound the right hand side of Equation (S20) by a simpler expression,

$$(\mathbf{x}_1 - \mathbf{x}_2)\,\mathbf{A}\left(\tanh([\mathbf{x}_1(t)]_+) - \tanh([\mathbf{x}_2(t)]_+)\right) \leq \|\mathbf{x}_1 - \mathbf{x}_2\|^2\,\|\mathbf{A}\|. \qquad \text{(S22)}$$

Now we assume $\|\mathbf{A}\| < \min(\lambda_V, \lambda_x)$ such that we can write $\lambda_V = \epsilon_V + \|\mathbf{A}\|$ and $\lambda_x = \epsilon_x + \|\mathbf{A}\|$ with $\epsilon_V > 0$ and $\epsilon_x > 0$. Using this in Equation (S19) yields

$$\begin{aligned}\frac{1}{2}\dot{\|\boldsymbol{\Delta}(t)\|^2} &= -\left(\epsilon_V + \|\mathbf{A}\|\right)(\mathbf{x}_1 - \mathbf{x}_2)\left([\mathbf{x}_1(t)]_- - [\mathbf{x}_2(t)]_-\right) \\ &\quad - \left(\epsilon_x + \|\mathbf{A}\|\right)(\mathbf{x}_1 - \mathbf{x}_2)\left([\mathbf{x}_1(t)]_+ - [\mathbf{x}_2(t)]_+\right) \\ &\quad + (\mathbf{x}_1 - \mathbf{x}_2)\,\mathbf{A}\left(\tanh([\mathbf{x}_1(t)]_+) - \tanh([\mathbf{x}_2(t)]_+)\right) \\ &= -\epsilon_V\,(\mathbf{x}_1 - \mathbf{x}_2)\left([\mathbf{x}_1(t)]_- - [\mathbf{x}_2(t)]_-\right) - \epsilon_x\,(\mathbf{x}_1 - \mathbf{x}_2)\left([\mathbf{x}_1(t)]_+ - [\mathbf{x}_2(t)]_+\right) \\ &\quad - \|\mathbf{A}\|\,\|\mathbf{x}_1 - \mathbf{x}_2\|^2 + (\mathbf{x}_1 - \mathbf{x}_2)\,\mathbf{A}\left(\tanh([\mathbf{x}_1(t)]_+) - \tanh([\mathbf{x}_2(t)]_+)\right), \qquad \text{(S23)}\end{aligned}$$

and together with Equation (S22)

$$\begin{aligned}\frac{1}{2}\dot{\|\boldsymbol{\Delta}(t)\|^2} &\leq -\epsilon_V\,(\mathbf{x}_1 - \mathbf{x}_2)\left([\mathbf{x}_1(t)]_- - [\mathbf{x}_2(t)]_-\right) - \epsilon_x\,(\mathbf{x}_1 - \mathbf{x}_2)\left([\mathbf{x}_1(t)]_+ - [\mathbf{x}_2(t)]_+\right) \\ &\quad - \|\mathbf{A}\|\,\|\mathbf{x}_1 - \mathbf{x}_2\|^2 + \|\mathbf{x}_1 - \mathbf{x}_2\|^2\,\|\mathbf{A}\| \\ &= -\epsilon_V\,(\mathbf{x}_1 - \mathbf{x}_2)\left([\mathbf{x}_1(t)]_- - [\mathbf{x}_2(t)]_-\right) - \epsilon_x\,(\mathbf{x}_1 - \mathbf{x}_2)\left([\mathbf{x}_1(t)]_+ - [\mathbf{x}_2(t)]_+\right). \qquad \text{(S24)}\end{aligned}$$

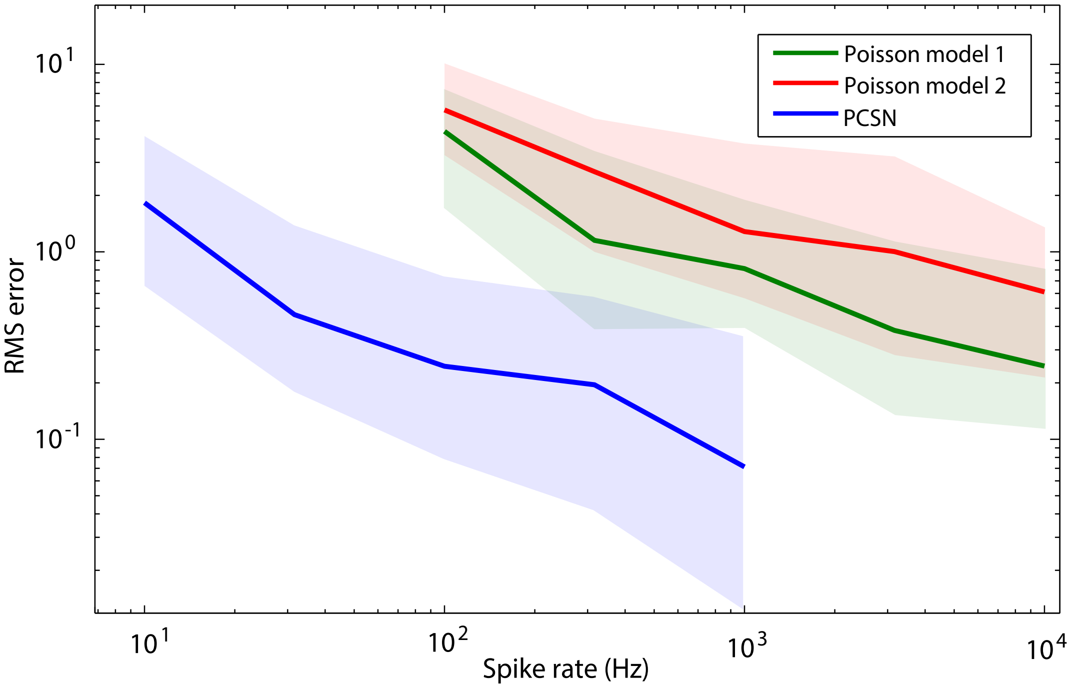

Figure A: Comparison of PCSNs and Poisson coding learning networks. *Error of PCSNs and Poisson coding learning networks with different spike rates after learning continuous dynamics. The panel shows the median error to the saw tooth target pattern (cf. Fig. 3d) during testing, in equidistant bins of the network spike rate (shaded: intervals between first and third quartile). The PCSN with its deterministic spike code reaches the same error level as the networks with simple Poisson coding with almost two orders of magnitude fewer spikes.*

Both terms on the right hand side are smaller or equal to zero,

$$(\mathbf{x}_1 - \mathbf{x}_2)\left([\mathbf{x}_1(t)]_\pm - [\mathbf{x}_2(t)]_\pm\right) = \left([\mathbf{x}_1(t)]_\pm - [\mathbf{x}_2(t)]_\pm\right)^2 - [\mathbf{x}_1(t)]_\pm [\mathbf{x}_2(t)]_\mp - [\mathbf{x}_2(t)]_\pm [\mathbf{x}_1(t)]_\mp \geq 0. \tag{S25}$$

We can therefore set $\epsilon = \min(\epsilon_V, \epsilon_x) > 0$ and simplify

$$\begin{aligned} \frac{1}{2}\,\|\dot{\mathbf{\Delta}}(t)\|^2 &\leq -\epsilon\,(\mathbf{x}_1 - \mathbf{x}_2)\left([\mathbf{x}_1(t)]_- - [\mathbf{x}_2(t)]_-\right) - \epsilon\,(\mathbf{x}_1 - \mathbf{x}_2)\left([\mathbf{x}_1(t)]_+ - [\mathbf{x}_2(t)]_+\right) \\ &= -\epsilon\,\|\mathbf{\Delta}(t)\|^2, \end{aligned} \tag{S26}$$

which is equivalent to

$$\|\dot{\mathbf{\Delta}}(t)\| \leq -\epsilon\,\|\mathbf{\Delta}(t)\|. \tag{S27}$$

The distance between different trajectories thus decreases at least exponentially fast with rate ϵ, for any input. We may conclude that $\|\mathbf{A}\| < \min(\lambda_V, \lambda_x)$ provides a sufficient condition for the system to possess the echo-state property.

1.3 Comparison of PCSNs with Poisson coding learning networks

In the following, we compare the performance of PCSNs and Poisson coding learning networks. To enable a direct comparison, we use PCSNs with saturating synapses and Poisson coding networks of the same size and with the same learning rule for the recurrent synapses such that in the high-rate limit, both network types become equivalent to the same continuous networks. As a specific task for the comparison, we choose learning of a saw tooth-like signal as displayed in Fig. 3d. We find

that both networks perform better for higher rates. However, due to their deterministic, precise spike code, the PCSNs achieve the same error levels with almost two orders of magnitude smaller rates, cf. Fig. A. This is generally a consequence of the fact that the population coding error in precisely spiking networks is much smaller than in Poisson coding networks, ref. [3] shows it to be proportional to $1/N$ (where N is the number of neurons in the network), while a simple Poisson population code has precision $1/\sqrt{N}$. However, in our PCSNs we have additional learning whose consequences on the precision of the output signal are not easy to determine.

The Poisson models are setup as follows: We start with the continuous target dynamics Equation (6) and for simplicity consider $\lambda_x = \lambda_V$ and $\gamma = \theta$, i.e.

$$\dot{x}_n(t) = -\lambda_V x_n(t) + \sum_{m=1}^{N} A_{nm} \tanh([x_m(t)]_+) + I_{\mathrm{e},n}(t). \tag{S28}$$

The state of the corresponding Poisson unit n shall be characterized by $u_n(t)$; we aim at $u_n(t) \approx x_n(t)$ for high spike rates. For the spike generation, we orient at standard models (e.g. [8]) and at keeping the dynamical Equations simple.

As Poisson model 1, we use networks of units with threshold and nonlinear saturation, specifically unit n has the rate

$$\nu_n(t) = s_0 \tanh\left([u_n(t)]_+\right). \tag{S29}$$

The constant s_0 allows to modulate the rate without changing the dynamics of $\mathbf{u}$. Given $\nu_n(t)$, the unit generates an inhomogeneous Poisson spike train $s_n(t)$ (cf. Equation (1)) with this rate. The spike train in turn generates postsynaptic inputs with decay time constant λ_s, as given in Equation (2). When rescaled with λ_s/s_0, the postsynaptic inputs satisfy for large s_0

$$\frac{\lambda_s}{s_0} r_n(t) \approx \frac{\nu_n(t)}{s_0} = \tanh\left([u_n(t)]_+\right). \tag{S30}$$

A network with dynamics

$$\dot{u}_n(t) = -\lambda_V u_n(t) + \sum_{m=1}^{N} A_{nm} \frac{\lambda_s}{s_0} r_m(t) + I_{\mathrm{e},n}(t) \tag{S31}$$

then approximates the continuous dynamics Equation (S28).

As Poisson model 2, we use networks of linear threshold units,

$$\nu_n(t) = s_0 \left[u_n(t)\right]_+.$$

They yield for large s_0

$$\frac{\lambda_s}{s_0} r_n(t) \approx [u_n(t)]_+ \tag{S32}$$

and

$$\dot{u}_n(t) = -\lambda_V u_n(t) + \sum_{m=1}^{N} A_{nm} \tanh\left(\frac{\lambda_s}{s_0} r_m(t)\right) + I_{\mathrm{e},n}(t) \tag{S33}$$

for the network dynamics approximating Equation (S28). We note that this model also satisfies a decoding Equation analogous to Equation (9), $\frac{\lambda_s}{s_0} r_n(t) \approx [x_n(t)]_+$.

We change the threshold θ (PCSNs) and the base rates s_0 (Poisson networks) to generate networks

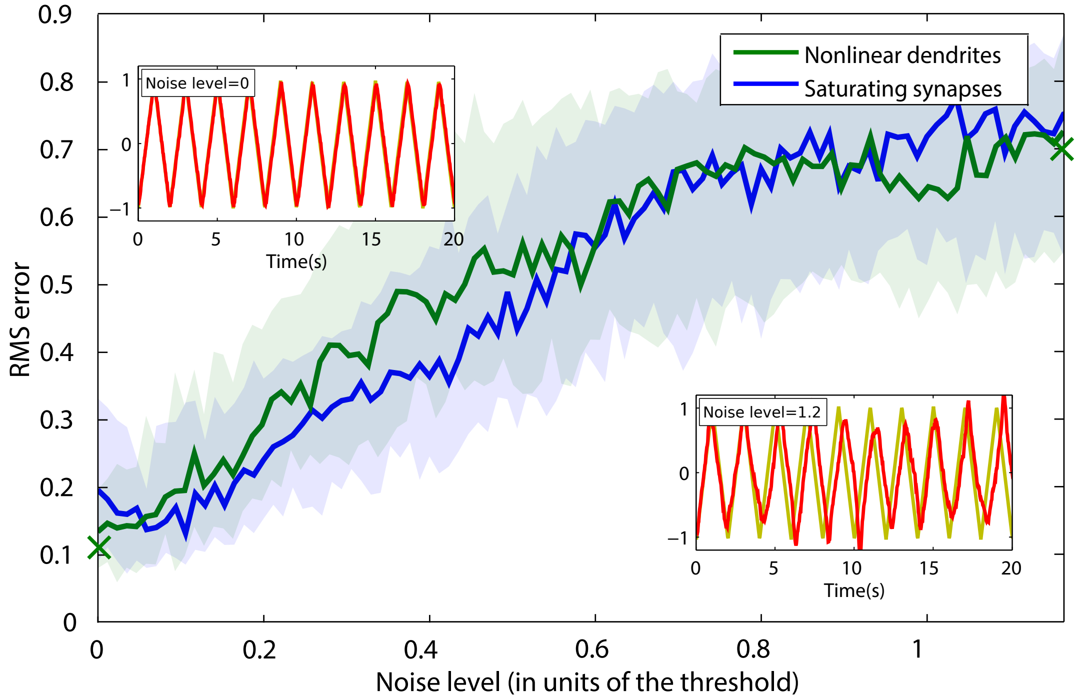

Figure B: Robustness of PCSNs against noise. *The figure shows the median RMS error between the output of PCSNs and the saw tooth target pattern during testing, versus the noise level (shaded: intervals between first and third quartile). The noise level is given in terms of the standard deviation generated by a purely noise-driven subthreshold membrane potential (Ornstein-Uhlenbeck process) with membrane time constant λ_V, in multiples of the threshold. The insets display the testing phase for two examples using the setup with nonlinear dendrites (crosses in the main plot denote the corresponding noise and error levels).*

with different rates. For PCSNs the remaining parameters are adapted such that the corresponding continuous network is also given by Equation (S28). For each parameter value we train 75 networks with different random topology and different initial conditions. We thereafter compute the actually generated average spike rates within log-scale equidistant bins. Further, we compute the root mean squared (RMS) error between the desired signal and the signal generated during testing. Fig. A displays the median and the first and third quartiles of the error versus the average rate in double logarithmic scale.

1.4 Robustness against noise

PCSN learning is robust against noise. Fig. B shows this by example of the learning of the saw tooth pattern (cf. Figs. 3d, A), both for PCSNs with saturating synapses and nonlinear dendrites. In each time step of the Euler-Maruyama integration, Gaussian noise is added. The noise level is given in terms of the standard deviation generated by a purely noise-driven subthreshold membrane potential (Ornstein-Uhlenbeck process) with membrane time constant λ_V, in multiples of the threshold (which equals $\theta/2$ in the case of saturating synapses and θ in the case of non-linear dendrites), i.e. noise-level $:= \frac{\sigma_\eta}{\sqrt{2\lambda_V}} \frac{\sqrt{(1-e^{-2})}}{\text{threshold}}$. The error is determined as RMS error between the desired signal and the signal generated during testing.

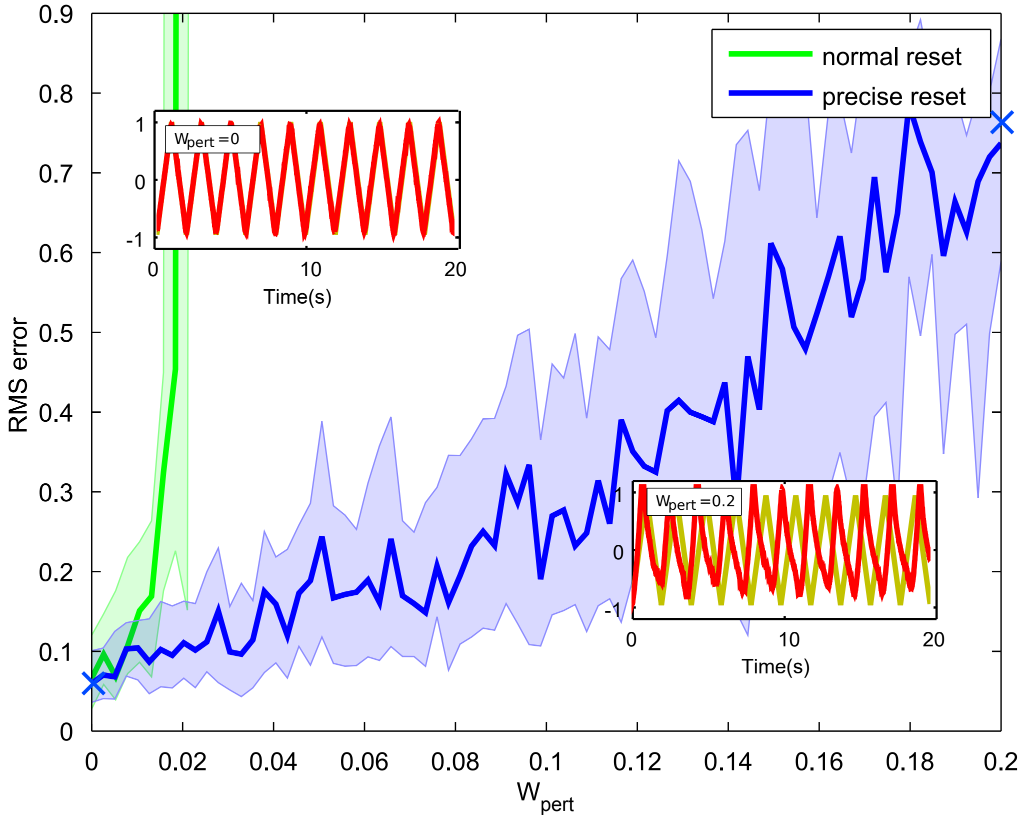

Figure C: Robustness of PCSNs against structural perturbations of dendritic coupling. *The figure shows performance of native PCSNs (networks without precise reset, green) and for PCSNs where neurons are reset to a fixed membrane potential after spike generation (networks with precise reset, blue). Displayed is the median RMS error between the output of PCSNs and the saw tooth target pattern during testing versus the standard deviation* W_{pert} *of a multiplicative Gaussian perturbation in the connectivity (shaded: intervals between first and third quartile). The insets display the testing phase for two examples using the setup with precise reset (crosses in the main plot denote the corresponding* W_{pert} *and error levels). While the native model does not show successful learning for perturbations larger than* 2% *of the connection weights, networks with precise reset generate a recognizable sawtooth pattern as learned output even for perturbations of* 20% *(inset at lower right). The error generated by networks with precise reset increases gradually with perturbation strength, while there is an abrupt change for the native networks.*

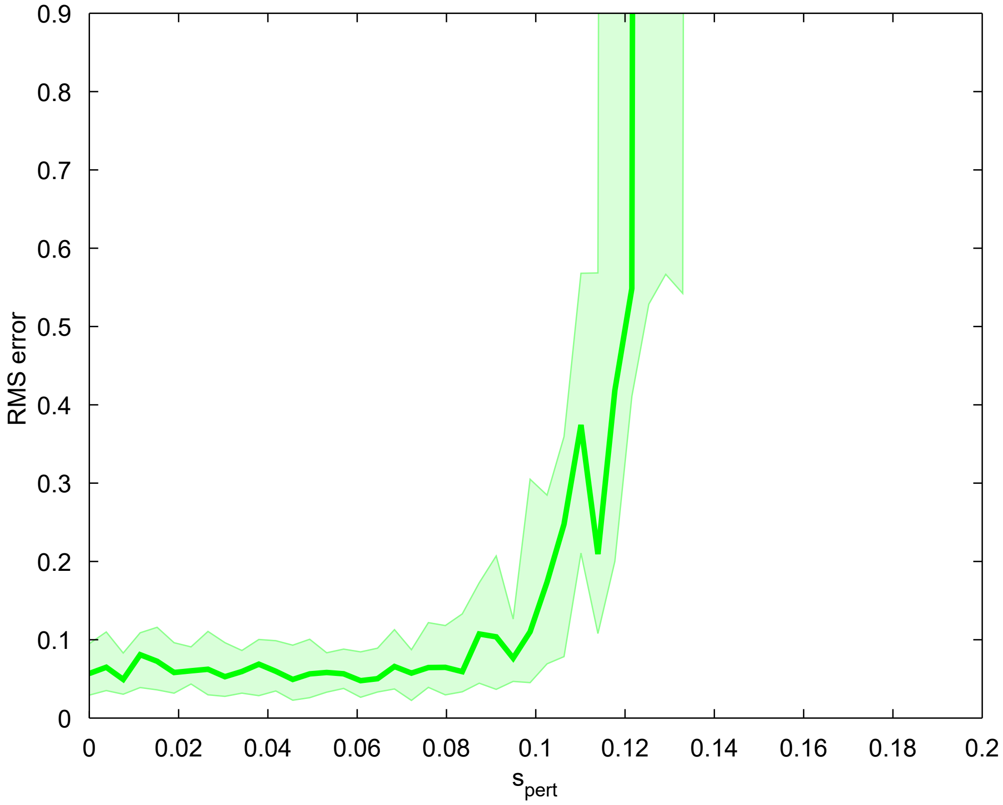

Figure D: Robustness of PCSNs against reduction of fast couplings. *The panel shows the median RMS error between the output of PCSNs and the saw tooth target pattern during testing versus the size s_{pert} of the multiplicative reduction of the fast connections (shaded: intervals between first and third quartile). For $s_{pert} < 10\%$ the error increases only slightly with increasing s_{pert}. A further increase of s_{pert} leads to a strong, rapid increase in the error and the PCSN is soon not able to learn the pattern anymore.*

1.5 Robustness of PCSNs with nonlinear dendrites against structural perturbations

In CSNs with nonlinear dendrites, the optimal strengths of the couplings from other neurons to the nonlinear dendrites and the fast couplings are independent of the parameters of the encoded nonlinear dynamical system Equation (S17). Since deviations from the optimal values in these couplings in general do not imply a simple change in the encoded dynamics but impair the coding scheme, we here investigate robustness of PCSN-learning against them.

The optimal coupling strength from neuron m to the jth nonlinear dendrite of neuron n is $W_{njm} = \Gamma_{jm}$. Here we test the robustness of the learning scheme against deviations from the optimal couplings. We find that PCSN learning is robust, if we modify the neuron model to have a "precise reset", i.e. the reset is always to the fixed value $-\theta$ (-2θ below threshold θ), even if fast excitation to a suprathreshold potential caused the spike (Fig. C). In the native model the reset has fixed size -2θ such that the membrane potential would be reset to a value larger than $-\theta$ after a suprathreshold excitation. We note that the reset to a fixed value may also be biologically more plausible than a reset of fixed size.

Fig. C shows this by example of the learning of the saw tooth pattern (cf. Figs. 3d, A). The W_{njm} are perturbed proportionally to the strength of their optimal values, the perturbed couplings are given by $W_{njm} = \Gamma_{jm}\left(1 + W_{\text{pert}}\Xi^{n}_{jm}\right)$, where the Ξ^{n}_{jm} are independently drawn from a Gaussian distribution with mean zero and variance one. We adopt the interpretation of the PCSNs as networks with plastic recurrent connections: The outputs, the output weight updates, the inverse correlation matrices and the updates of the dendrite-to-soma weights D_{nj} are computed using Equations (14), (18), (19) and (22) with unperturbed readouts $\tilde{r}_j(t) = \tanh\left(\sum_{m=1}^{N}\Gamma_{jm}r_m(t)\right)$. We note that updating the D_{nj} is not equivalent to a static feedback of the updated overall network readout anymore, since the latter does not contain the perturbed W_{njm}.

Our findings raise the question why the networks with precise reset are much more stable to perturbations in the dendrites. We find that the instability in the simulations of the conventional model is due to an explosion of the spike rates, such that every neuron spikes once at every simulated time step. This is due to the "ping pong effect" already described in ref. [3]: Neurons that spike due to excitation from fast connections generate further suprathreshold excitation and in the end all neurons have spiked within a single step. In [3] this problem is solved using a higher value of μ. We find for our simulations with perturbed W_{njm} that a fine tuning of μ is required to prevent a breakdown of the learning. In contrast, the precise reset solves this problem robustly. This is a consequence of the fact that on the one hand the precise reset yields a membrane potential that is further away from the threshold and thus reduces the chance of re-excitation of a neuron that has recently spiked (μ has an in principle similar effect). On the other hand, the difference from the theory arises only after suprathreshold excitation, the change in the precise spiking dynamics is small and the spike coding scheme is preserved.

The optimal strength of a fast coupling from neuron m to neuron n is $U_{nm} = \sum_{j=1}^{J}\Gamma_{jn}\Gamma_{jm} + \mu\delta_{nm}$ (which is independent of N for fixed neuron threshold). In "balanced state" irregular spiking networks, such recurrent connections may be expected to be much smaller, since they scale with $1/\sqrt{N}$ for fixed neuron thresholds [9]. We therefore test the dependence of the PCSNs on these connections by a multiplicative weakening by a factor $1 - s_{\text{pert}}$, $U^{\text{pert}}_{nm} = (1 - s_{\text{pert}})U_{nm}$ for $n \neq m$ (resets are kept $U^{\text{pert}}_{nn} = U_{nn}$). Fig. D shows that PCSNs are robust against this: The learning capabilities are conserved, if $s_{\text{pert}} < 10\%$. The figure also indicates that the fast connections are important for PCSN

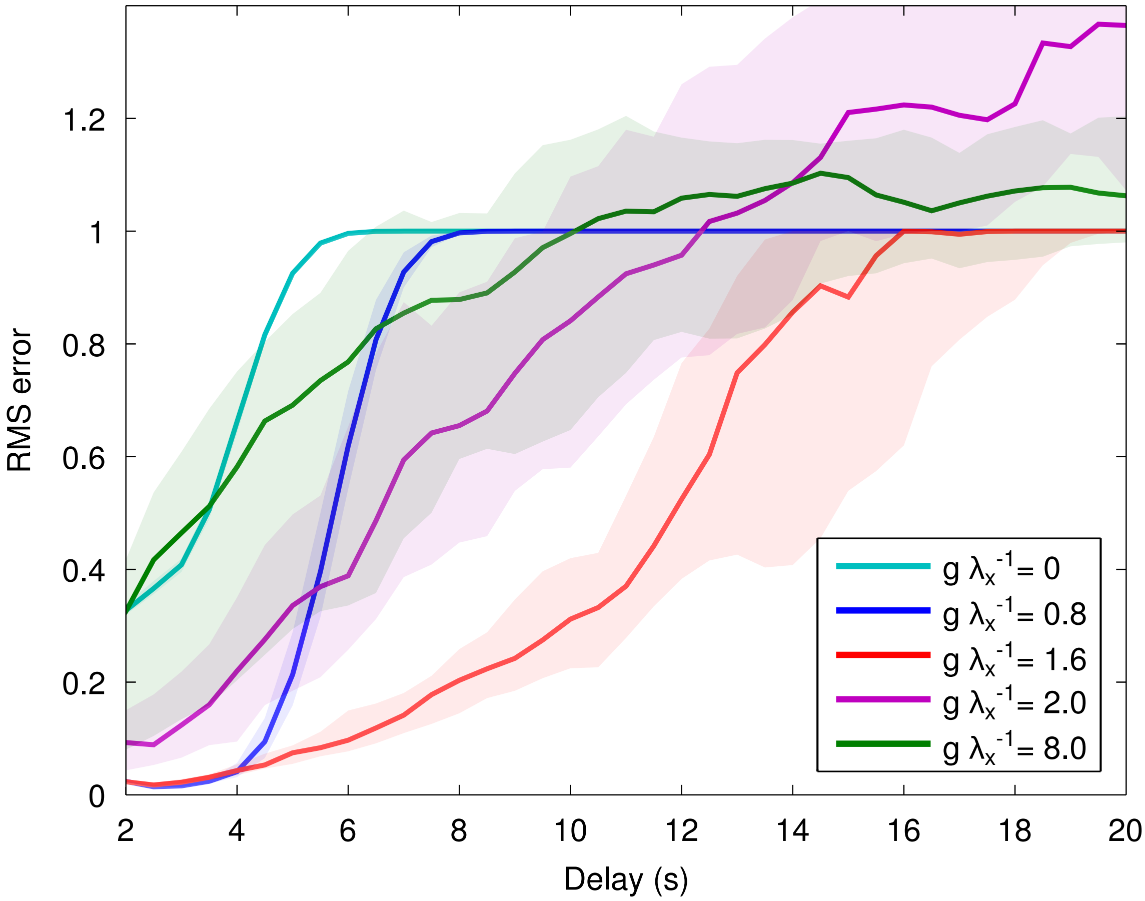

Figure E: Memory duration in CSNs with different recurrent coupling strengths. *Supporting figure to Fig. 4a-c, displaying a direct comparison between multiple error vs. reaction delay traces. A disconnected network, $g\lambda_x^{-1} = 0$, has comparably short memory. Increase of connection strength leads to an increase of memory duration (cf. the trace for $g\lambda_x^{-1} = 0.8$). Memory is most persistent around $g\lambda_x^{-1} = 1.6$, and decreases for larger coupling strengths, as expected for systems where the dynamics become more and more chaotic ($g\lambda_x^{-1} = 2$, $g\lambda_x^{-1} = 8$).*

functionality, since the learning abilities are quickly lost as s_{pert} increases beyond this range.

1.6 Comparison of network sizes and the spike rate-network size trade-off

We illustrate that for PCSNs, network sizes comparable to those of continuous rate networks solving the same task with FORCE learning can be sufficient. Further, we show that there is a trade-off between network size and spike rate of individual neurons. As example we use the "camel's hump" task (cf. Fig. 3e). Fig. Fa shows that continuous networks Equation (6) can learn the signal well for about $N > 40$, we use $N = 50$ as a reference. We compare with PCSNs with nonlinear dendrites that encode a system Equation (6) with the same parameters (in particular $J = 50$), and are trained to solve the same task. We compare the RMS error and spiking frequencies for different PCSN sizes N and γ_s, which regulates the threshold of the neurons (cf. Methods and Equation (S10)). Fig. Fb,c shows the trade-off between the number of neurons in the PCSNs and their individual spiking frequency: For $\gamma_s \approx 0.1 - 0.15$ only the networks with $N = 200$ and $N = 400$ learn the task reliably (panel (b)), the neurons adopt a mean spike rate of about or smaller 100Hz. For sufficiently

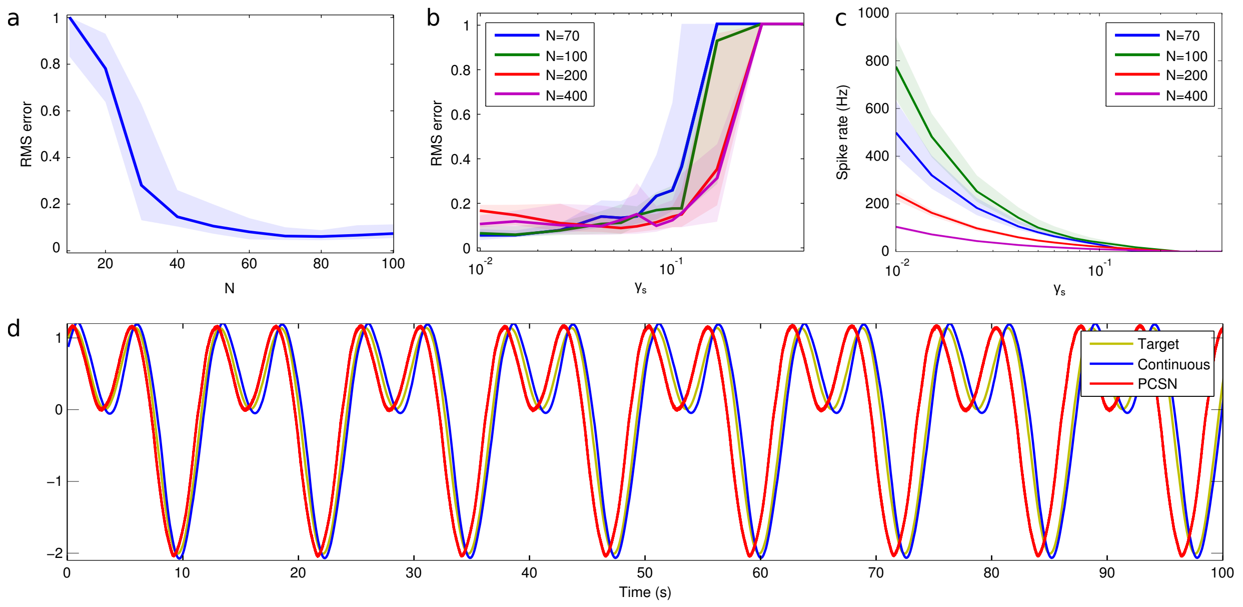

Figure F: Network size and PCSN spiking rate trade-off. *(a): RMS errors of continuous rate networks of different size, after training the camel's hump task (cf. Fig. 3e). The networks generate the pattern well for network sizes larger approximately* $N = 40$*. (b,c): RMS errors of PCSNs with nonlinear dendrites, after training the camel's hump task. The panels display the trade-off between network size and spike frequency: For* $N = 200$*, a small error can be achieved with a single neuron spike rate of* 100*Hz (cf. RMS error at* $\gamma_s \approx 0.1$ *displayed in (b) and rate at* $\gamma_s \approx 0.1$ *displayed in (c)). Networks with* $N = 400$ *need only a spike rate of* 10*Hz. Smaller networks (e.g.* $N = 70$*) need higher spike rates but reach the same error levels. (We note that for small networks we observe a nonmonotonic dependence of the spike rate on the network size for constant* γ_s*.) (d): Example dynamics. The PCSN signal (red trace,* $\gamma_s = 0.01$*,* $N = 70$*,* $J = 50$*) approximates the target function (yellow trace) similarly well as a continuous rate neuron network of similar size (blue trace,* $N = 50$*).*

small γ_s also smaller networks learn the task well, but all networks generate a higher spike frequency.

1.7 Error evolution for longer times

We do not observe lasting changes of the error in long term simulations. For periodic signals, there is an inevitable phase shift. It originates from the small error between the period of the desired and the learned signal; this error accumulates over time. Apart from that, tested features such as the deviation from the desired dynamics (RMS error) and the spike frequency are remarkably constant. Fig. Ga,b illustrates and quantifies this for the camel's hump task (Fig. 3e). Fig. Ga displays the continued desired dynamics and the occurring phase shift for longer recall durations. Fig. Gb shows that the RMS error is stationary, approximately constant over time, if one corrects for the shift. For the Lorenz attractor (Fig. 3f-h), the teacher and student trajectories quickly depart from each other after the end of learning. The spiking network nevertheless continues to generate dynamics that after decoding agree with the dynamics of a Lorenz system. Fig. Gc shows this for longer times. As a quantitative check, the tent map Fig. 3h relating subsequent local maxima in the z-coordinate shows the good agreement with the tent-map generated by the teacher dynamics also for long times. In the displayed simulation, the Lorenz dynamics deviate three times from the desired dynamics, which leads to the six outliers in Fig. 3h. However, the dynamics return every time to the desired dynamics such that the errors just generate single outlier pairs in the tent map and the qualitative dynamics agree with those of the Lorenz system still after 4000s.

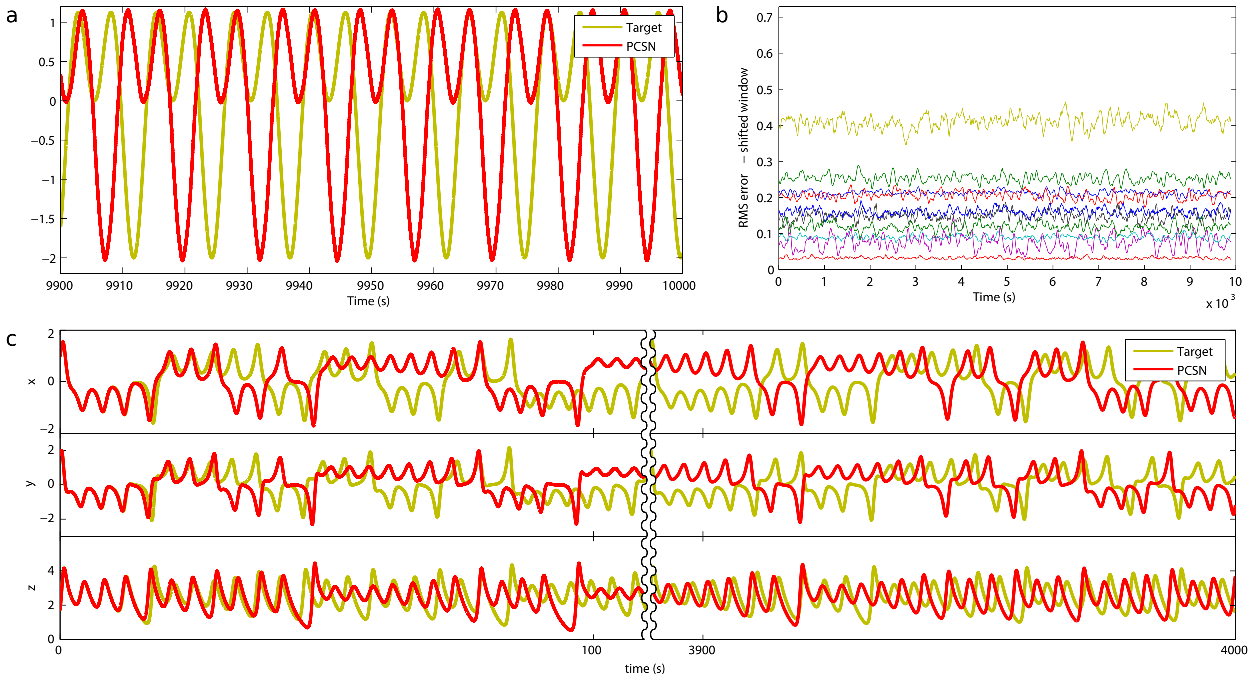

Figure G: Long term evolution. *(a): PCSN generated camels's hump signal and target (Figs. 3e, F) after long times: the signal is phase-shifted compared to the target but otherwise not noticeably changed. Panel (b) quantifies this observation by plotting the RMS error, corrected for a possible phase shift to the target, against time. Displayed are several learning trials with different random initial connectivity. The error is stationary, constant except for fluctuations. (c): Lorenz attractor signal of Fig. 3f-h, dynamics of x, y, z vs. time. Also after long times, the PCSN (red trace) in general generates qualitatively the same dynamics as the target Lorenz system (yellow trace).*

Sat. syn.	N	α	dt	T_t	λ_s^{-1}	λ_V^{-1}	V_r	θ
Fig. A (PCSN)	50	0.01	0.001ms	10.5s	100ms	1s	0.9θ	swept
Fig. B	100	0.1	0.1ms	30s	100ms	50ms	0.9θ	0.05

Table A: *Parameters used in the different figures for simulations of networks with saturating synapses.*

2 Supporting methods

2.1 Details on the supporting figures

The parameters of the different simulations are given in Table A for simulations using saturating synapses and in Table B for simulations using nonlinear dendrites. Further parameters and details about the figures and simulations are given in the following paragraphs.

If not mentioned otherwise, for all simulations we use $g = 1.5\frac{1}{\text{s}}$, $p = 0.1$, $\tilde{w}^f = 1\frac{1}{\text{s}}$, $\tilde{w}^i = 1\frac{1}{\text{s}}$, $\Delta t = 0.01\text{s}$, $\gamma = \theta$ and $\sigma_\eta = 0\frac{1}{\sqrt{\text{s}}}$.

Figure A

The signal has period 2s and amplitude 10. The parameters of the Poisson networks are $N = 50$, $\alpha = 0.01$, $g = 1.5\frac{1}{\text{s}}$, $T_t = 10.5\text{s}$, $\lambda_s^{-1} = 100\text{ms}$, $\lambda_V^{-1} = 1\text{s}$, $dt = 0.1/s_0$, the sparse matrix $\mathbf{A}$ has a fraction $p = 0.1$ of nonzero entries, which are drawn from a Gaussian distribution with zero mean and variance $\frac{g^2}{pN}$. s_0 is swept between $50\frac{1}{s}$ and $21544\frac{1}{s}$ (values in the different sweeps: $s_0 = 50\frac{1}{\text{s}}$, $100\frac{1}{\text{s}}$, $150\frac{1}{\text{s}}$, $200\frac{1}{\text{s}}$, $215\frac{1}{\text{s}}$, $464\frac{1}{\text{s}}$, $1000\frac{1}{\text{s}}$, $2150\frac{1}{\text{s}}$, $4641\frac{1}{\text{s}}$, $10000\frac{1}{\text{s}}$, $21544\frac{1}{\text{s}}$). The PCSN has saturating synapses.

Nonlin. dendr.	N	J	α	γ_s	dt	T_t	μ	λ_s^{-1}	λ_V^{-1}	a
Fig. B	200	100	0.1	0.05	0.1ms	30s	$1/N^2$	100ms	50ms	λ_s−1Hz
Fig. C	200	100	0.1	0.05	0.1ms	30s	$1/N^2$	100ms	50ms	λ_s−1Hz
Fig. D	200	100	0.1	0.05	0.1ms	30s	$1/N^2$	100ms	50ms	λ_s−1Hz
Fig. Fb, c	swept	50	1	swept	0.1ms	100.5s	0	100ms	100ms	λ_s−1Hz
Fig. Fd	70	50	1	0.01	0.1ms	100.5s	0	100ms	100ms	λ_s−1Hz
Fig. Ga, b	70	50	1	0.01	0.1ms	100.5s	0	100ms	100ms	λ_s−1Hz

Table B: *Parameters used in the different figures for simulations of networks with nonlinear dendrites. The parameter $a = \lambda_s - \lambda_x$ is given in terms of λ_s and λ_x*

θ is swept between 0.5 and 0.01 (specific values of the sweep: θ =0.5, 0.4, 0.3, 0.2, 0.1, 0.09, 0.08, 0.07, 0.06, 0.05, 0.04, 0.03, 0.02, 0.01). We compute the RMS error between the signal and the target in the first 10.5s after training. The error is computed using a normalized version of the signal, where the amplitude is set to 1.

Figure B

The signal has period 2s and amplitude 15 (normalized to one in the figure). We take medians and quartiles over 50 trials. The noise level is given in terms of the standard deviation generated by a purely noise-driven subthreshold membrane potential (Ornstein-Uhlenbeck process) with membrane time constant λ_V, in multiples of the threshold (which equals $\theta/2$ in the case of saturating synapses and θ in the case of non-linear dendrites), i.e. noise-level := $\frac{\sigma_\eta}{\sqrt{2\lambda_V}} \frac{\sqrt{(1-e^{-2})}}{\text{threshold}}$. Plotted are the median of the RMS error (shaded: intervals between first and third quartile) between the signal and the target in the first 30s after training. The error is computed using the normalized version of the signal, where the amplitude is set to 1. The sweep covers 101 equidistant values of σ_n from $0\frac{1}{\sqrt{s}}$ to $0.01\frac{1}{\sqrt{s}}$ in the case of non-linear dendrites and 101 equidistant values of σ_n from $0\frac{1}{\sqrt{s}}$ to $0.2\frac{1}{\sqrt{s}}$ in the case of saturating synapses.

Figure C

The signal has period 2s and amplitude 10 (normalized to one in the figure). We take medians and quartiles over 50 trials. We use the Euler method to integrate the differential Equations.

Plotted are the median of the RMS error (shaded: intervals between first and third quartile) between the signal and the target in the first 30s after training. The error is computed using the normalized version of the signal, where the amplitude is set to 1. The sweep covers 80 equidistant values of W_{pert} from 0 to 0.2.

For simulations that showed pathological spiking (more than 200 spikes per time step in the numerical simulation) we assigned an infinite error.

Figure D

The signal has period 2s and amplitude 10 (normalized to one in the figure). We take medians and quartiles over 50 trials. We use the Euler method to integrate the differential Equations.

Plotted are the median of the RMS error (shaded: intervals between first and third quartile) between the signal and the target in the first 30s after training. The error is computed using the normalized

version of the signal, where the amplitude is set to 1. The sweep covers 80 equidistant values of s_{pert} from 0 to 0.3 (displayed is the range up to 0.2).

For simulations that showed pathological spiking (more than 200 spikes per time step in the numerical simulation) we assigned an infinite error.

Figure E

The parameters are as in Fig. 4a-c, lower panels, see "Figure details" in the main text.

Figure F

Fig. Fa: The continuous networks obey Equation (11) (Equation (S17)), they are endowed with the FORCE learning rule. The parameters of the network are $\lambda_x = 1\frac{1}{s}$, $dt = 0.01$s, and the sparse matrix $\mathbf{A}$ has a fraction $p = 0.1$ of nonzero entries, which are drawn from a Gaussian distribution with zero mean and variance $\frac{g^2}{pN}$ with $g = 1.5\frac{1}{s}$. The task from Fig. 3e serves as target signal. The learning rate is $\alpha = 1$, the learning time is $T_t = 100.5$s. The network size N is swept from 10 to 100 in steps of 10. The figure shows the median of the RMS error (shaded: intervals between first and third quartile) between the signal and the target in the first 10s after training. The statistics are based on 100 trials per value of N.

Fig. Fb,c: We use PCSNs with nonlinear dendrites, which encode continuous dynamics as generated by the rate networks in panel (a). γ_s is swept over $0.4, 0.25, 0.15, 0.1, 0.09, 0.075, 0.06, 0.05, 0.04, 0.025, 0.015, 0.01$ and networks of size N are plotted for $N = 70, 100, 200, 400$. Fig. Fb shows the median of the RMS error (shaded: interval between first and third quartile) between the signal and the target in the first 10s after training. Fig. Fc shows the median of the mean spike rate per neuron (shaded: intervals between first and third quartile). The statistics are based on 20 trials per parameter combination.

Fig. Fd: The plot shows example patterns generated by continuous networks as used in panel (a) (blue trace) and PCSNs as used in panels (b,c) (red trace). For the continuous network $N = 50$, for the PCSNs $\gamma_s = 0.01, N = 70, J = 50$. The PCSN has a mean spike rate of 441 Hz

Figure G

Fig. Ga: The panel shows the last 100s of the PCSN simulation displayed in Fig. Fd. Total duration of the recall phase is 10000s, Fig. Fd shows the first 100s.

Fig. Gb: We use the same network parameters as in Fig. Fd and Fig. Ga, displayed are 10 learning trials with different randomly chosen initial connectivity. We compute a phase-shift corrected version of the RMS error in a sliding window of size 100s, for different starting points τ of the sliding window. τ is in the range from 1s to 10000s with step size 1s. The phase-shift corrected version of the RMS error is computed by $\sqrt{\frac{1}{100\text{s}}\int_{\tau}^{\tau+100\text{s}}\left(\text{signal}(\widetilde{t}) - \text{target}(\widetilde{t}+\Delta)\right)^2 d\widetilde{t}}$ with the phase-shift Δ of the target signal chosen such that the integral is minimal.

Fig. Gc: The parameters are the same as in Fig. 3h.

Supporting references

[1] Boerlin M, Denève S (2011) Spike-based population coding and working memory. PLoS Comput Biol 7: e1001080.

[2] Bourdoukan R, Barrett DG, Machens CK, Denève S (2012) Learning optimal spike-based representations. Advances in Neural Information Processing Systems 25: 2294-2302.

[3] Boerlin M, Machens CK, Denève S (2013) Predictive coding of dynamical variables in balanced spiking networks. PLoS Comput Biol 9: e1003258.

[4] Jaeger H, Haas H (2004) Harnessing nonlinearity: Predicting chaotic systems and saving energy in wireless communication. Science 304: 78-80.

[5] Sussillo D, Abbott LF (2009) Generating coherent patterns of activity from chaotic neural networks. Neuron 63: 544–557.

[6] Lukosevicius M, Jaeger H, Schrauwen B (2012) Reservoir computing trends. Künstl Intell 26: 365–371.

[7] Jaeger H (2001) The "echo state" approach to analysing and training recurrent neural networks-with an erratum note. Bonn, Germany: German National Research Center for Information Technology GMD Technical Report 148: 34.

[8] Gerstner W, Kistler W (2001) Spiking Neuron Models: Single Neurons, Populations, Plasticity. Cambridge: Cambridge Univ. Press.

[9] Renart A, de la Rocha J, Bartho P, Hollender L, Parga N, et al. (2010) The asynchronous state in cortical circuits. Science 327: 587-590.
